# Hydrogel-based 3D human iPSC-derived neuronal culture for the study of rabies virus infection

**DOI:** 10.3389/fcimb.2023.1215205

**Published:** 2023-08-25

**Authors:** Papon Muangsanit, Thanathom Chailangkarn, Nathiphat Tanwattana, Ratjika Wongwanakul, Porntippa Lekcharoensuk, Challika Kaewborisuth

**Affiliations:** ^1^ Virology and Cell Technology Research Team, National Center for Genetic Engineering and Biotechnology (BIOTEC), National Science and Technology Development Agency (NSTDA), Pathumthani, Thailand; ^2^ Interdisciplinary Program in Genetic Engineering and Bioinformatics, Graduate School, Kasetsart University, Bangkok, Thailand; ^3^ National Nanotechnology Center (NANOTEC), National Science and Technology Development Agency (NSTDA), Pathumthani, Thailand; ^4^ Department of Microbiology and Immunology, Faculty of Veterinary Medicine, Kasetsart University, Bangkok, Thailand; ^5^ Center for Advance Studies in Agriculture and Food, KU Institute Studies, Kasetsart University, Bangkok, Thailand

**Keywords:** human-induced pluripotent stem cells, neurons, hydrogels, culture model, rabies virus, NanoString, gene expression, virus-host interaction

## Abstract

**Background:**

Rabies is a highly fatal infectious disease that poses a significant threat to human health in developing countries. In vitro study-based understanding of pathogenesis and tropism of different strains of rabies virus (RABV) in the central nervous system (CNS) is limited due to the lack of suitable culture models that recapitulate the complex communication pathways among host cells, extracellular matrices, and viruses. Therefore, a three-dimensional (3D) cell culture that mimics cell-matrix interactions, resembling in vivo microenvironment, is necessary to discover relevant underlying mechanisms of RABV infection and host responses.

**Methods:**

The 3D collagen-Matrigel hydrogel encapsulating hiPSC-derived neurons for RABV infection was developed and characterized based on cell viability, morphology, and gene expression analysis of neuronal markers. The replication kinetics of two different strains of RABV [wild-type Thai (TH) and Challenge Virus Standard (CVS)-11 strains] in both 2D and 3D neuronal cultures were examined. Differential gene expression analysis (DEG) of the neuropathological pathway of RABV-infected 2D and 3D models was also investigated via NanoString analysis.

**Results:**

The 3D hiPSC-derived neurons revealed a more physiologically interconnected neuronal network as well as more robust and prolonged maturation and differentiation than the conventional 2D monolayer model. TH and CVS-11 exhibited distinct growth kinetics in 3D neuronal model. Additionally, gene expression analysis of the neuropathological pathway observed during RABV infection demonstrated a vast number of differentially expressed genes (DEGs) in 3D model. Unlike 2D neuronal model, 3D model displayed more pronounced cellular responses upon infection with CVS-11 when compared to the TH-infected group, highlighting the influence of the cell environment on RABV-host interactions. Gene ontology (GO) enrichment of DEGs in the infected 3D neuronal culture showed alterations of genes associated with the inflammatory response, apoptotic signaling pathway, glutamatergic synapse, and trans-synaptic signaling which did not significantly change in 2D culture.

**Conclusion:**

We demonstrated the use of a hydrogel-based 3D hiPSC-derived neuronal model, a highly promising technology, to study RABV infection in a more physiological environment, which will broaden our understanding of RABV-host interactions in the CNS.

## 1 Introduction

Rabies is an acute, almost invariably fatal form of viral encephalomyelitis in humans caused by rabies virus (RABV). The transmission of the virus occurs through contact with the saliva of infected animals, typically via bites, scratches, or exposure to the mucous membrane (Fooks et al., 2017). Rabies poses an enormous threat to human health in developing countries as well as rural regions, with an annual death toll of approximately 59,000 people worldwide (Schnell et al., 2010; CDC, 2020). Basic knowledge of RABV and the physio-pathological mechanisms involved in rabies encephalitis is ultimately needed for the development of effective novel treatment modalities such as antiviral drugs.

Despite extensive and progressive studies, many aspects of virus pathogenesis and host responses in relation to RABV and host interaction remain uncertain due to the diverse range of cell types, virus titers, and virus strains employed in various experimental infections resulting in different outcomes. While human pathology and animal studies have been instrumental in identifying regions of CNS affected by RABV infection, the development of *in vitro* models is often critical for the discovery of immunological and virological mechanisms that lead to CNS diseases. Most of the *in vitro* data currently available for RABV infections are from studies in monolayer cultures. Although the findings acquired from two-dimensional (2D) cell cultures have broadened knowledge regarding RABV-host interactions, these culture models lack some of the intricate biological processes appear *in vivo* (Jensen and Teng, 2020).

Studies have reported that cells respond differently to three-dimensional (3D) environments compared with 2D settings (Loessner et al., 2010). 3D cell culture is more representative of native tissues in terms of cell-matrix interactions, matrix composition, protein concentration gradients, stiffness, and mechanical stresses (Duval et al., 2017). A previous study demonstrated that the proportion of neurons and astrocytes infected with the RABV was similar in the 3D *ex vivo* mouse brain tissues, whereas the majority of infected cells were neurons in 2D culture (Potratz et al., 2020), indicating the necessity of 3D CNS culture to discover relevant underlying mechanisms of RABV infection and host responses.

A range of approaches are available for 3D model development including spontaneous cell aggregation (spheroid and organoid), scaffold-based culture systems, micro-carrier beads as well as microfluidic 3D cell culture (Zhuang et al., 2018). In contrast to scaffold-free organoid systems, scaffold-based systems offer a microenvironment that closely mimics physiological conditions, facilitating enhanced biophysical and biochemical interactions between cells and the extracellular matrix (ECM) (Tupone et al., 2021). While Matrigel-based 3D organoids have been widely employed in virology research, their reproducibility is often inconsistent, and their generation can be costly (Kim et al., 2021). Currently, hydrogels, which are crosslinked 3D networks of hydrophilic polymer chains, have been developed as particularly promising materials for 3D scaffold-based culture because of their high biocompatibility, tunable biodegradability, and porous structures (Lam et al., 2020; Vallejo-Giraldo et al., 2020).

Interactions between 3D environments and viruses have also been shown to more closely reflect viral replication and transmission within physiological target tissues (Imle et al., 2019). The hydrogel platform offers a simpler setup, greater consistency, and the ability to tailor mechanical and chemical properties of the culture model. Moreover, the controlled seeding of specific cell populations into hydrogels enables the examination of cellular responses in particular cell types, a capability that is not achievable with multicellular organoids.

Hydrogels have been widely used to generate 3D CNS-like neuronal models, but a few of them has been employed in virological studies. ECM-collagen hydrogels have been demonstrated to support primary human neuron viability and differentiation for up to 30–32 days *in vitro* (Lam et al., 2020). In addition, Raimondi et al. (2020) have developed collagen-polyethylene glycol hydrogels embedded with primary mouse cortical neurons which formed a complex neuronal network (Raimondi et al., 2020).

In rabies research, the use of hiPSC-derived neurons to investigate RABV infection and cellular responses has been recently established (Sundaramoorthy et al., 2020; Chailangkarn et al., 2021). Although the 2D culture of hiPSC-derived neurons provides an unlimited resource to explore the RABV infection in cells of human origin, this model still lacks cell-matrix and virus-matrix interactions present *in vivo*.

Here, we developed a 3D neuronal culture model using hiPSC-derived neurons and collagen-Matrigel hydrogel blends to study RABV infection. Growth kinetics of RABV strains were compared in both 2D and 3D formats. Differential gene expressions of neuropathological pathways in response to the different RABV strains in 2D and 3D cultures were examined. We have initiated the utilization of 3D hiPSC-derived neurons as a promising *in vitro* platform resembling RABV infection *in vivo* to investigate RABV and host interaction at molecular levels. We anticipate that this model will help generate progressive knowledge on RABV infection.

## 2 Materials and methods

### 2.1 Cells and viruses

HiPSCs used in this study were kindly provided by Dr. Methichit Wattanapanitch (Siriraj Center for Regenerative Medicine (SiCRM), Research Department, Faculty of Medicine Siriraj Hospital, Mahidol University, Bangkok, Thailand) (Netsrithong et al., 2019). The use of hiPSCs was approved by the Institutional Review Board (IRB) Ethics Committee of the National Science and Technology Development Agency (NSTDA) (NIRB-038-2562). The protocol for the derivation of neural progenitor cell (NPC) to neurons has been described previously (Chailangkarn et al., 2021). Neuronal differentiation was initiated by the removal of fibroblast growth factor 2 (FGF2) (Sigma, MO, USA) from NPC media consisting of DMEM/F12 (HyClone, UT, USA), 0.5× N2 (Invitrogen, MA, USA), 0.5× B27 (Invitrogen), 1× GlutaMAX (Thermo Fisher Scientific, MA, USA), 1× antibiotic-antimycotic (Thermo Fisher Scientific). The neuronal culture was fed every 3–4 days for at least 2–3 weeks.

BHK21-C13 cells (ATCC CCL-10™) were grown in Dulbecco’s Modified Eagle’s Medium (DMEM) (Thermo Fisher Scientific). Media were supplemented with 10% v/v heat-inactivated fetal bovine serum (FBS; Thermo Fisher Scientific) and 1% v/v Penicillin/Streptomycin (Thermo Fisher Scientific). All cell cultures and subsequent experiments were kept in a humidified incubator with 5% CO_2_ at 37°C.

A wild-type Thai RABV strain (TH), which was isolated from a rabid dog’s brain, was obtained from Queen Saovabha Memorial Institute (Bangkok, Thailand). The virus was propagated in BHK21 cells to increase virus titer. The RABV challenge virus standard-11 (CVS-11) strain, a laboratory reference strain, was kindly provided by professor Pongrama Ramasoota (Center of Excellence for Antibody Research (CEAR), Faculty of Tropical Medicine, Mahidol University). Two different RABV strains were used for comparative studies of virus growth and host responses to RABV infection in neurons.

### 2.2 Hydrogel fabrication

#### 2.2.1 Collagen hydrogel

To prepare 1 ml of collagen gel, 100 µl of 10× MEM (Sigma-Aldrich) was mixed with 800 µl of type 1 rat tail collagen (3 mg/ml in 0.6% acetic acid; Thermo Fisher Scientific) and the mixture was neutralized using 43 µl of 1 N sodium hydroxide (RCI Labscan, Bangkok, Thailand) before addition of 100 µl of cell suspension (culture medium containing 0.2–2.0 × 10^6^ cells/ml as required). For multi-well plate assays, hydrogels were cast by adding 60 µl and 400 µl of the mixture to individual wells of 96-well plates and 24-well plates, respectively. The plates were placed in an incubator at 37°C, 5% CO_2_ for 30 min to allow the hydrogels to set.

#### 2.2.2 Collagen-Matrigel hydrogel blend

Collagen hydrogels were fabricated in the same way as above, at a 1:8 ratio of 10× MEM and collagen type I. Collagen-Matrigel blends were prepared at ratios of 1:1.25 and 1:2.50 mg/ml of collagen to Matrigel (Corning, NY, USA) by protein concentration. Pure collagen was also prepared as a control. Hydrogel blends were cast into multi-well plates and placed in an incubator at 37°C, 5% CO_2_ for 30 min to set.

### 2.3 2D and 3D neuronal cultures

NPCs were differentiated in 2D culture to achieve 10-day hiPSC-derived neurons. The presence of cortical layer-specific neurons expressing the early-developed deep layer markers CTIP2 and postsynaptic density protein 95 (PSD95) was verified via immunofluorescence staining (**Supplementary Figure 1**) before seeding into the hydrogel scaffolds. Pre-differentiated neuronal cells were then dissociated using Accutase™ cell detachment solution (Stemcell Technologies) and gently resuspended in medium. 2D neuronal cultures were prepared in 24-well plates at a density of 1×10^5^ cells per well while 3D cultures were prepared at a density of 1×10^6^ cells per 1 ml of hydrogel (400 µl per well in 24-well plates). The medium was changed every 2–3 days and cells remained in culture for 1–21 days depending on the experiments. For virus infection, hiPSC-derived neurons were cultured for a week before virus adsorption.

### 2.4 Live-dead staining

Live-dead cell staining was performed at various time points (24–168 hours) after cell seeding. Hoechst-33342 (Invitrogen) dissolved in phosphate-buffered saline (PBS) and propidium iodide (PI) solution (Thermo Fisher Scientific) were added directly to the cell culture medium at final concentrations of 2 μg/ml and 5 μg/ml, respectively. Cells were incubated for 1 hour and then immediately examined with an IX83 fluorescence microscope (Olympus). For each sample, three regions of at 20x magnification were randomly selected for quantification with ImageJ software (Arshadi et al., 2021).

### 2.5 WST-1 metabolic activity assay

Metabolic activity in neuron cultures was measured at various time points (24–168 hours) after cell seeding. Cell-embedded hydrogels were prepared in 96-well plates. Ten microliters of cell proliferation reagent WST-1 (Promega, WI, USA) were added into each well. Plates were incubated for 2 hours at 37°C, 5% CO_2_. Absorbance was measured at 450 nm in a Synergy HTX microplate reader (BioTek, CA, USA). Each sample was prepared in biological triplicate and three technical replicates.

### 2.6 Quantitative RT-PCR

The 2D and 3D samples were rinsed with PBS before harvesting. Cells were collected using TRIzol™ reagent (Invitrogen). Total RNA was isolated using Direct-Zol™ RNA Microprep kits (Zymo Research, CA, USA). Sample RNAs were eluted in 35 µl of DNase/RNase-free water, of which 10 µl per sample were analyzed by real-time quantitative PCR (RT-qPCR) using the Luna Universal One-Step RT-qPCR kit (New England Biolabs, MA, USA). Each sample was prepared in biological triplicate and two technical replicates and reactions were run using the CFX96 Touch Real-Time PCR Detection System (Bio-Rad, CA, USA). The housekeeping gene β-actin was used as an internal control for data normalization. The delta-delta Ct (ΔΔCt) algorithm was used to calculate relative changes in gene expression (Livak and Schmittgen, 2001).

### 2.7 Virus infection

Neuronal cultures were adsorbed with TH or CVS-11 at a multiplicity of infection (MOI) of 0.5 for 2 hours. Cells were washed twice with PBS before adding fresh media. Cell supernatants were collected at indicated time points for viral growth curve analysis.

For virus titration, BHK21-C13 cells were grown in 96-well plates. Fifty microliters of ten-fold dilutions of infectious cell supernatant were transferred into each well of the 96-well plate in quadruplicate. The cells were incubated at 37°C for 1 hour. After incubation, supernatants were removed and 100 µl of fresh Opti-MEM™ media (Thermo Fisher Scientific) were added to each well. At 72 hours post-infection (hpi), cells were fixed with 80% cold acetone for 5 mins and washed with PBS plus 0.05% Tween-20 (PBST). Horse α-rabies hyperimmune serum raised against RABV TH (Queen Saovabha Memorial Institute) was added to each well to probe for RABV proteins. Dylight488-conjugated goat α-horse IgG (Abcam, Cambridge, UK) was used as the secondary antibody. Virus titer (TCID_50_/_mL_) was calculated using the Reed and Muench method (Reed and Muench, 1938).

### 2.8 Immunofluorescence

The 2D and 3D samples were fixed in 4% paraformaldehyde for 30 mins at 4 °C and washed twice with PBS for 10 mins. Cells were then permeabilized with 0.5% Triton X-100 (Sigma-Aldrich) diluted in PBS for 30 mins at room temperature and washed three times with PBS. Cells were blocked with 3% bovine serum albumin (BSA) (HiMEDIA, PA, USA) for 30 mins at room temperature. After the blocking step, appropriate primary antibody dilutions of rabbit α-MAP2 (Abcam; ab183830), rabbit α-β-III tubulin (Abcam; ab18207), rat α-CTIP2 (Abcam; ab 18465), mouse α-PSD95 (Novus Biologicals; nb300-556) and horse α-rabies antiserum were added and incubated overnight at 4°C. The following day, gels were washed six times with PBS (5 mins per each round) and incubated with secondary antibodies, namely Alexa Fluor^®^ 568-conjugated goat α-rabbit (Invitrogen) and Dylight 488-conjugated goat α-horse IgG (Abcam; ab102391), at 1:250 dilutions. Alexa Fluor 488 Phalloidin (Thermo Fisher Scientific) was also used. Cells were stained with Hoechst-33342 and visualized under an IX83 fluorescence microscope (Olympus).

### 2.9 Confocal microscopy and image analysis

Neuron morphological characteristics in 2D and 3D cultures were analyzed using Leica SP8 confocal microscopy (Leica, Wetzlar, Germany). Three z-stacks were randomly selected from the middle region of each gel. An imaging volume of 424.85 µm **×** 424.85 µm **×** 104.36 µm with a voxel size of 0.244 µm × 0.244 µm × 4.348 µm (20x magnification) was captured and flattened into a single z-stack. For each experiment, the same volume and number of z-stacks were taken, and the same acquisition settings were used. The length of neurites was analyzed using the NeuronJ plugin in the ImageJ software.

### 2.10 Analysis of gene transcripts via NanoString

#### 2.10.1 RNA extraction

Total RNA was isolated using TRIzol™ following the manufacturer’s instructions. RABV-infected 2D and 3D samples were collected at 8 and 24 hpi. In 24-well plates, 2D samples were homogenized in 500 µl of TRIzol™ and 3D samples were homogenized in 1,000 µl of TRIzol™ by pipetting up and down several times until the homogenates appeared consistent with no visible clumping. RNA from TRIzol™-lysed samples was purified using the Direct-zol™ RNA Microprep kit, treated with DNase I, eluted in 35 µl RNase-free water, and stored at -80°C until used. The quality of the purified RNA was measured using the Nanodrop 2000 spectrophotometer (Thermo Fisher Scientific). Gel electrophoresis was run to confirm there was no cellular DNA contamination of the purified total RNA.

#### 2.10.2 Neuropathology transcript analysis

One hundred nanograms of purified total RNA were analyzed using the nCounter Analysis System (NanoString Technologies, WA, USA), according to the manufacturer’s instructions. Differential gene expression of neurons grown in 2D and 3D formats was examined using the nCounter^®^ Neuropathology panels (NanoString Technologies) which are composed of 770 unique probe pairs for associated human genes to evaluate neurodegenerative and neuropathogenesis pathways following RABV infection. The assay is based on direct detection of targets using molecular barcodes, without the necessity of reverse transcription and mRNA amplification.

Purified total RNA samples were analyzed using the NanoString nCounter XT gene expression assay following the manufacturer’s protocol. Briefly, 5 µl of 50 ng of total RNAs were mixed with 2 μl of the Capture Probe set and 8 µl of hybridization buffer containing the Reporter CodeSet and mixtures were allowed to hybridize by incubation at 65°C in a thermal cycler for 16 hours. Post-hybridization processing and data collection were performed using the nCounter Analysis System.

Raw data were pre-processed using the nSolver™ 4.0 software according to nCounter gene expression data analysis guidelines. Data pre-processing included sample quality control (QC), background and normalization. Probes against 14 external RNA control consortiums (ERCC) consisted of 6 synthetic DNA positive and 8 negative controls. The geometric mean of positive control counts in each sample was applied to QC to evaluate overall assay efficiency, assay linearity and limit of detection (LOD). A correlation value higher than 0.95 indicated high assay efficiency (**Supplementary Figure 2**). Negative controls were used to estimate non-specific binding of probes within the samples being run and background correction. Reference housekeeping genes were used for data normalization according to the nSolver™ 4.0 software.

### 2.11 Gene ontology analysis

Differential gene expression (DEG) between RABV-infected neurons versus uninfected controls, TH versus CVS-11, and 2D versus 3D models in each time point was evaluated by applying cutoffs at a fold-change (FC) of 1.5 (0.58log_2_FC for upregulated genes and -0.51log_2_FC for downregulated genes). Functional enrichment, GO and pathway analyses of up- and downregulated DEGs of RABV-infected neurons in 2D and 3D settings were performed using the over-representation analysis method (ORA) of WebGestalt (Wang et al., 2013) and the STRING database (http://string-db.org).

### 2.12 Nitric oxide production assay

The 2D and 3D neuronal models were prepared in 24-well plates and inoculated with TH and CVS-11 at an MOI of 0.5. At 24 hpi, cell supernatants were collected and nitric oxide concentrations were measured using a Griess reagent assay kit (R&D Systems, MN, USA) to quantify the amount of nitrite and nitric oxide metabolites produced by the nitric reductase reaction. Absorbance was measured at 540 nm using a Synergy HTX microplate reader and analyzed via the Gen5 software.

### 2.13 Caspase-3 ELISA assay

2D and 3D neuronal models were prepared in 24-well plates and inoculated with TH and CVS-11 at an MOI of 0.5. At 24 hpi, cell pellets were collected for measurement of active caspase-3 concentrations using the human-cleaved Caspase-3 ELISA kit (Abcam; ab220655) according to the manufacturer’s protocol. Absorbance was measured at 450 nm using a Synergy HTX microplate reader and analyzed via the Gen5 software.

### 2.14 Statistical analyses

Statistical analyses of data were performed using GraphPad Prism 5.0 (GraphPad Software Inc, CA, USA). Prior to statistical testing, Shapiro-Wilk normality tests were used to determine whether repeats have a Gaussian distribution. For comparisons of experiments with two parameters, Student’s t-tests were performed for statistical significance and standard errors (SEM) were calculated. For experiments with multiple treatments for comparison, one-way ANOVA tests (or Kruskal-Wallis tests for non-parametric distribution) or two-way ANOVA were performed, accompanied by multiple comparison tests. Data shown in all graphs are mean ± SEM unless otherwise stated. Differences were considered significant when *p* < 0.05. For all statistics, data from at least three independent samples or repeated experiments were used.

## 3 Results

### 3.1 Optimization and characterization of 3D neuronal culture

**Figure 1:**
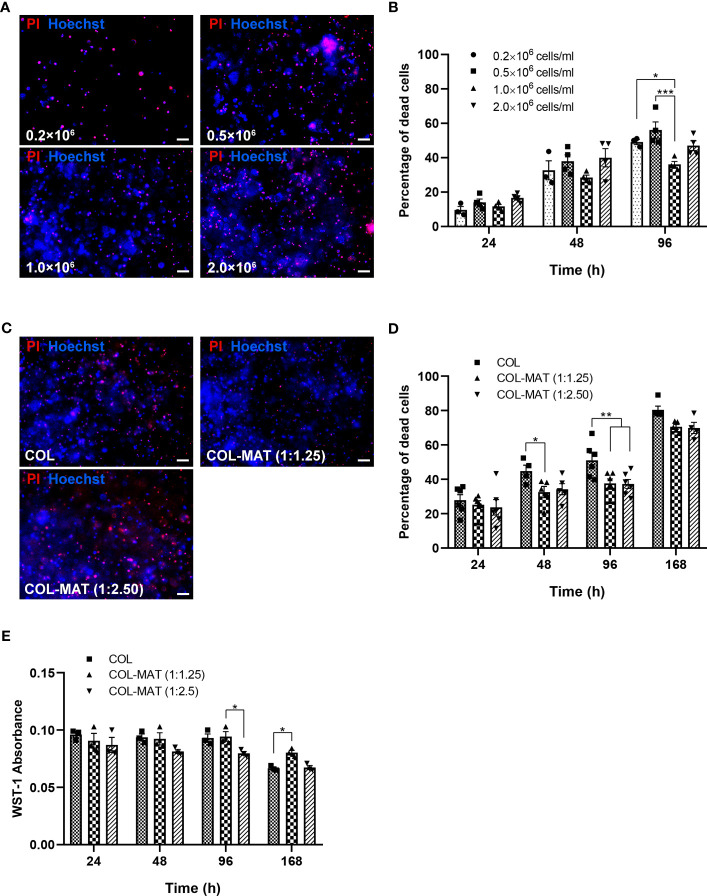
Effect of cell seeding density and matrix composition on viability of hiPSC-derived neurons. **(A, B)** HiPSC-derived neurons were seeded in 1 mg/ml collagen hydrogels in 96-well plates at four different cell seeding densities. Cells were treated with PI and Hoechst stains at specified time points. **(A)** Fluorescence images were captured by fluorescence microscopy. Representative images of PI (red) and Hoechst (blue) staining at 96 hours are shown. Scale bars = 50 µm. **(B)** The percentage of dead cells was calculated by counting manually using ImageJ of the proportion of PI-stained cells to Hoechst-stained cells for three randomly selected fields of view (20x). Data are presented as mean ± SEM; *n =* 3-4. **(C, D)** HiPSC-derived neurons were seeded in collagen hydrogels and collagen-Matrigel hydrogel blends at the density of 1×10^6^ cells/ml. Cells were treated with PI and Hoechst stains at specified time points. **(C)** Fluorescence images were captured by fluorescence microscopy. Representative images of PI (red) and Hoechst (blue) staining at 168 hours are shown. Scale bars = 50 µm. **(D)** The percentage of dead cells was calculated by counting manually using ImageJ of the proportion of PI-stained cells to Hoechst-stained cells for three randomly selected fields of view (20x). Data are represented as mean ± SEM*; n =* 4-6. **(E)** The metabolic activity was determined based on absorbance values of the WST-1 assay. Data are represented as mean ± SEM; *n* = 3. *p<0.05; **p<0.01; ***p<0.001; COL, collagen; MAT, matrigel.

To develop a 3D neuronal culture model, cell densities and hydrogel compositions were first investigated. Cell seeding density is one of the most important factors for developing an optimal *in vitro* model because of its impact on cell survival and cell interactions (Issa et al., 2011; Coy et al., 2020). The viability of hiPSC-derived neurons at various densities ranging from 0.2×10^6^ to 2.0×10^6^ cells/ml of collagen gel was compared using propidium iodide (PI)/Hoechst staining. Representative fluorescence images of neurons showed less PI staining for 1.0×10^6^ cells/ml seeding density at 96 hours (**Figure 1A**), suggesting low amounts of dead cells. Cell deaths gradually increased over 96 hours for all densities (**Figure 1B**). However, neurons at 1.0×10^6^ cells/ml showed the lowest percentage of dead cells throughout the time points (**Figure 1B**). A cell seeding density of 1.0×10^6^ cells/ml was deemed optimal for further experiments.

A previous study showed that Matrigel could enhance neural stem cell viability in collagen mixtures (Vagaska et al., 2020). We thus examined the effect of different collagen-Matrigel blends in improving the viability of iPSC-derived neurons. Two ratios of collagen-Matrigel blends (1:1.25 and 1:2.5 mg/ml) substantially reduced cell death compared to a pure collagen hydrogel, suggesting the potency of Matrigel to promote cell survival (**Figures 1C, D**). Metabolic activity of neurons in all hydrogel conditions were reduced at 168 hours (Day 7), with higher metabolic activity in the 1:1.25 mg/ml collagen-Matrigel (**Figure 1E**) suggesting more cell proliferation compared to other conditions. The Matrigel alone group was not included since the Matrigel (2 mg/ml) did not completely form and gradually became soft and dissolved after cell culturing.

**Figure 2:**
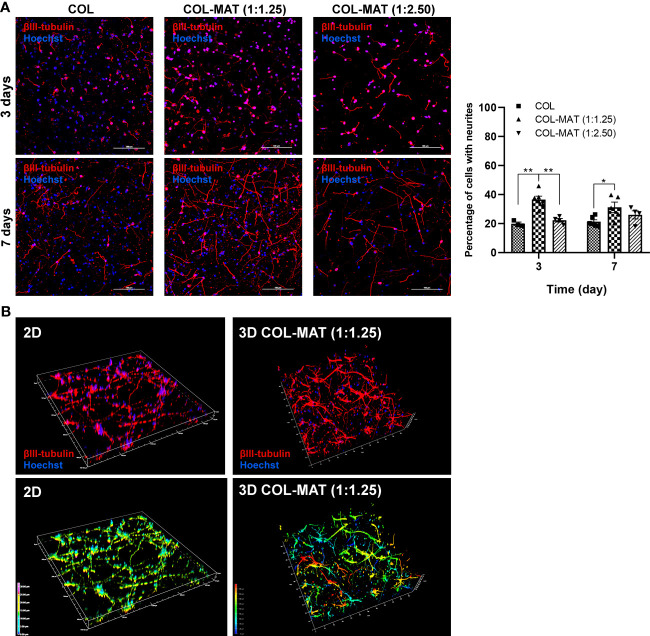
Comparison of neurite formation between 3D collagen-Matrigel blends and pure collagen. **(A)** HiPSC-derived neurons were seeded in collagen hydrogels and collagen-Matrigel hydrogel blends at the density of 1×10^6^ cells/ml. Cells were stained with β-III tubulin and Hoechst at specified time points. Representative confocal z-stack micrographs at day 3 and day 7 of hiPSC-derived neurons within collagen hydrogels and collagen-Matrigel hydrogel blends were captured. Forty to fifty z-stack images were taken at 2 µm intervals. Scale bars = 100 µm. The percentage of cells with neurites was calculated by counting manually using ImageJ of the proportion of β-III tubulin-stained cells to Hoechst-stained cells for three randomly selected fields of view (20x) with approximately 150 cells per field of view. Data are represented as mean ± SEM; *n* = 3-6. *p<0.05; **p<0.01. **(B)** Representative 3D reconstructed confocal images of neurons in 2D and 3D collagen-Matrigel blends at day 7 of culture. Colors indicate the depth of the cells in the culture model along the given z-axis; COL, collagen; MAT, matrigel.

To determine whether the collagen-Matrigel blends supported neurite formation, the numbers of neurons with neurites were calculated. Percentage of neurons with neurites in 1:1.25 collagen-Matrigel (mean ± SD of 33.86 ± 3.79%) was greater than that in a pure collagen format (mean ± SD of 20.83 ± 0.65%) (**Figure 2A**). Confocal 3D reconstructions revealed a 3D interconnected neuronal network within the 3D hydrogels (color-coded by the depth of penetration) compared to the more restricted planar 2D monolayer culture (**Figure 2B**). In addition, hiPSC-derived neurons in 2D culture tended to aggregate as cluster-like structures whereas more dispersed and connected neurons were observed in 3D culture (**Supplementary Figure 3**).

**Figure 3:**
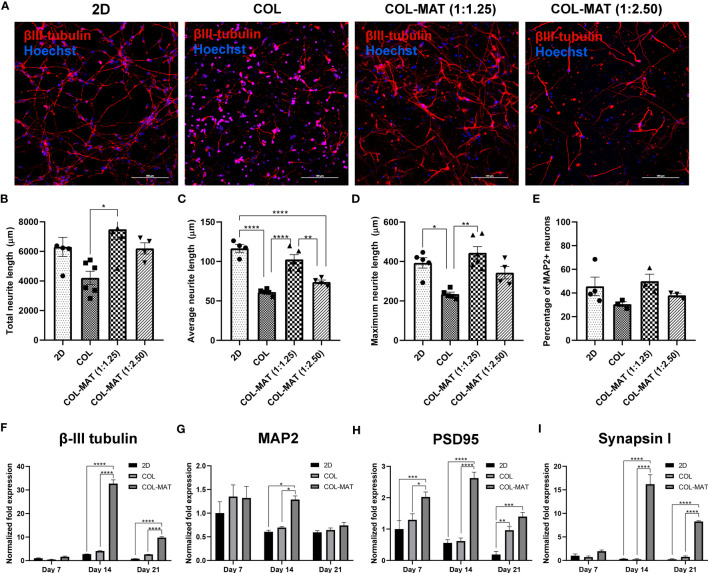
Impact of collagen-Matrigel on neuronal differentiation, maturation and synapse formation. **(A)** HiPSC-derived neurons were seeded in 2D monolayer, 3D collagen hydrogels and 3D collagen-Matrigel hydrogel blends at the density of 1×10^6^ cells/ml. Cells were stained with β-III tubulin and Hoechst at day 7. Representative confocal micrographs of β-III tubulin-positive cells (red). Scale bars = 100 µm. The morphological parameters of neurons including **(B)** total neurite length, **(C)** average neurite length, and **(D)** maximum neurite length was quantified using NeuronJ plugin in ImageJ for three randomly selected fields of view (20x) for each experimental batch with approximately 150 cells per field of view. **(E)** A graph showing the percentage of MAP2-positive neurons calculated per fields of view (20x). Data are represented as mean ± SEM; *n* = 4-6. **(F–I)** HiPSC-derived neurons were seeded in 2D monolayer, 3D collagen hydrogels and 3D 1:1.25 mg/ml collagen-Matrigel hydrogel blends at the density of 1×10^6^ cells/ml. Total RNA was isolated at indicated time points. Graphs present the fold change of β-III tubulin, MAP2, PSD95, and Synapsin1 gene expression by RT-qPCR. Fold-changes were expressed by normalizing to their respective control, 2D culture at day 7. Data are represented as mean ± SEM; *n* = 3. *p<0.05; **p<0.01; ***p<0.001; ****p<0.0001; COL, Collagen; MAT, Matrigel.

We then assessed whether the 3D hydrogel scaffold accelerates neuronal differentiation, maturation, and synapse formation using specific neuronal markers. Neuronal morphology was evaluated using the NeuronJ Plugin in ImageJ software for the following parameters: total neurite length, average neurite length per neuron, maximum neurite length, and percentages of MAP2-positive cells per field of view (20x). Collagen-Matrigel blends yielded comparable total neurite lengths as the 2D culture and significantly higher values than pure collagen (**Figures 3A, B**). HiPSC-derived neurons in collagen hydrogels had a shorter neurite length compared to collagen-Matrigel blends after 7 days of culture (**Figures 3C, D**), with neurons embedded in the 1:1.25 mg/ml collagen-Matrigel showing notably greater neurite lengths compared to other hydrogels. Similar proportions of MAP2-positive neurons were observed across different hydrogel conditions with relatively lower numbers in the collagen group (**Figure 3E**).

The neuronal structural marker β-III tubulin displayed strong expression after 14 days of neural differentiation within the 1:1.25 mg/ml collagen-Matrigel blend but declined (~4 fold) after 21 days (**Figure 3F**). However, neurons cultured in 2D and collagen hydrogels had lower β-III tubulin expression at all time points. Expression levels of β-III tubulin in the collagen-Matrigel blend were higher than in 2D culture (~8 fold) at day 21, suggesting robust neuronal differentiation in the 3D format. The mature neuronal marker MAP2 was more highly upregulated in collagen-Matrigel than in the 2D model and collagen alone at day 14 (**Figure 3G**). At day 21, MAP2 expression level in collagen-Matrigel dropped to levels comparable to the other conditions. The post-synaptic marker PSD 95, which is a major protein in the excitatory synapses of the brain, exhibited greater levels of expression in collagen-Matrigel at all time points despite reduced expression levels at day 21 (**Figure 3H**). The pre-synaptic marker Synapsin1, which functions in regulating axon elongation and synaptic vesicle fusion, was more highly expressed in the collagen-Matrigel blend at day 14 than in 2D culture and collagen hydrogel (**Figure 3I**). Altogether, 3D culture demonstrated robust support for neuronal growth, exhibiting the highest neuronal gene expression at 14 days and sustaining higher gene expressions compared to 2D model for up to 21 days inferring that 3D culture would be a suitable model for conducting a several-week growth curve analysis.

### 3.2 Growth kinetics of RABV in a 3D neuronal model

**Figure 4:**
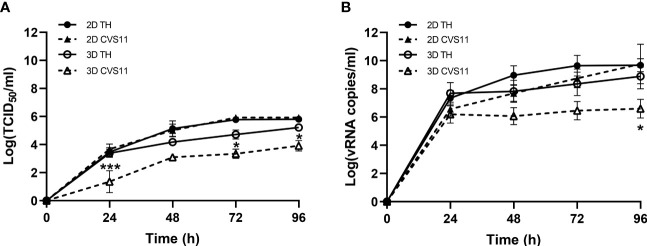
Growth kinetics of RABV infection in hiPSC-derived neurons cultured in 2D and 3D models. Neurons seeded in 2D or 3D culture were infected with TH or CVS-11 at an MOI of 0.5. Cell supernatants were collected daily for **(A)** virus titration and **(B)** vRNA analysis by RT-qPCR. Data are represented as mean ± SEM; *n* = 3. *p<0.05; ***p<0.001.

Having achieved an optimal 3D neuronal culture model, we next investigated whether the embedded neurons were permissive to RABV infection compared to the conventional 2D culture model. Replication kinetics of two different RABV strains, TH and CVS-11, were examined. Culture supernatants were collected at indicated time points and subjected to virus titration and vRNA measurement. Both strains of RABV exhibited comparable growth kinetics in 2D culture while TH infectious titers and vRNA were higher at all time points in 3D culture, with significant differences as compared to the CVS-11-infected samples (**Figures 4A, B**), indicating that sophisticated 3D culture may have an impact on host-virus interactions.

### 3.3 Differential gene expression in RABV-infected 2D and 3D neuronal cultures

**Figure 5:**
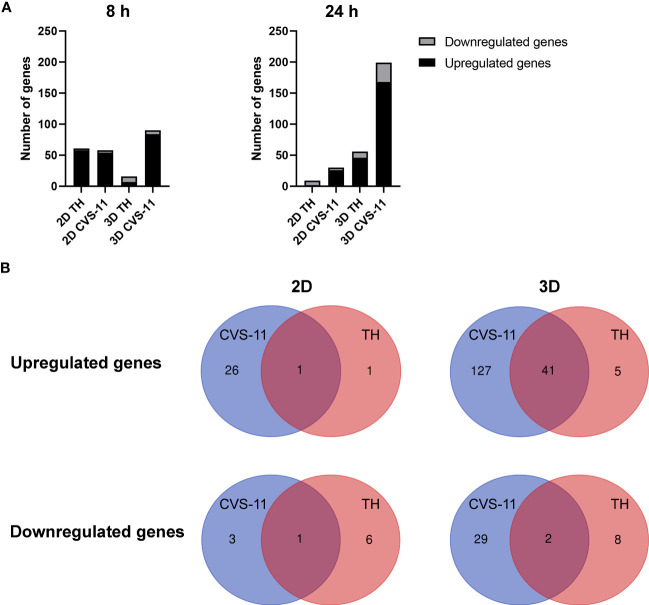
Differential gene expression from NanoString analysis of RABV-infected 2D and 3D hiPSC-derived neuronal models. **(A)** HiPSC neurons seeded in 2D and 3D cultures were infected with CVS-11 or TH at an MOI of 0.5. At 8 hpi and 24 hpi, cells were harvested for RNA isolation. The purified RNA was subjected to NanoString analysis and data normalization. Upregulated and downregulated genes were identified at a threshold of ≥ 1.5-fold change in expression levels. **(B)** DEGs of RABV-infected neurons in 2D and 3D models were classified for CVS-11 (blue) and TH (red) infection. Numbers in the overlapping regions indicate common DEGs.

To determine whether changes in gene expression could explain the differences in virus growth kinetics between the culture models, we next performed a gene expression analysis of the RABV infected hiPSC-derived neurons in both 2D and 3D model. Neurons seeded in 2D or 3D culture were infected with TH or CVS-11 at an MOI of 0.5. At 8 and 24 hpi, infected cells were collected for RNA isolation. The purified RNA was subjected to NanoString analysis. Normalized data were used for differential gene expression (DEG) analysis of RABV-infected hiPSC-derived neurons in both 2D and 3D models. DEG between RABV-infected neurons versus uninfected controls, TH versus CVS-11, and 2D versus 3D models in each time point was evaluated by applying cutoffs at a fold-change (FC) of 1.5 (0.58log2FC for upregulated genes and -0.51log2FC for downregulated genes). Overall, genes associated with neuropathological pathways were differentially expressed in the two models. Noticeably, stronger cellular responses were observed in mock-infected and infected neurons grown in 3D (**Supplementary Figure 4**), with DEGs listed in **Supplementary File 1**. The number of DEGs of neurons in 2D culture infected with different RABV strains were comparable, but substantially higher for CVS-11-infected neurons in 3D (**Figure 5A**), especially at 24 hpi.

In the 2D neuronal model, fewer upregulated and downregulated genes were observed for both RABV strains (CVS-11: 27 upregulated and 4 downregulated genes; TH: 2 upregulated and 7 downregulated genes). On the other hand, a total of 168 genes were found to be upregulated and 31 genes downregulated in the 3D neuronal model infected with CVS-11 while 46 upregulated and 10 downregulated genes were observed for TH infection (**Figure 5B** and **Supplementary Files 2, 3**). After comparing gene expression between the RABV strains, we found more commonly upregulated genes (41 genes) between the two strains in the 3D model compared to only 1 gene in the 2D model (**Figure 5B** and **Supplementary File 4**), suggesting that neurons grown in 3D respond more broadly to RABV infection. Many distinct genes were induced in RABV-infected 3D neuronal culture compared to 2D culture (**Figure 5B** and **Supplementary File 4**), with CVS-11 inducing a wider range of genes compared to TH (**Figure 5B** and **Supplementary File 4**).

Many of the differentially upregulated and downregulated genes detected in our 3D model have also been described by several previous studies of RABV-infected human and animal brain tissues (**Tables 1**, **2**), suggesting that our 3D hiPSC-derived neuronal model could mimic the *in vivo* environment. Commonly expressed genes found in both 2D and 3D culture models and distinct genes found in infected 3D but not in infected 2D culture models are shown in **Tables 1**, **2**, respectively.

**Table 1:** Previously identified proteins or genes in RABV-infected *in vivo* tissue samples and genes expressed in both 2D and 3D RABV-infected hiPSC-derived neuronal models.

Genes expressed in both 2D and 3D culture models					
Reported proteins/genes in RABV-infected *in vivo* samples			Related genes found in RABV-infected hiPSC-derived neuronal model		
Protein/gene	Expression change (reference)	Sample	Gene	GO: ID-Term name	Expression change
**Antidiuretic hormone (ADH)**	Increase (Preuss et al., 2009) Increase (Ferguson and Roll, 1981)	Highly pathogenic silver-haired bat-associated rabies virus (SB strain)-infected mouse brain Human serum	AVP	GO:2001233- regulation of apoptotic signaling pathway GO:0003013- circulatory system process	Upregulation
**ICAM1**	Increase (Long et al., 2020)	GD-SH-01-infected mouse brain	ICAM1	GO:0002376- immune system process GO:0001618- virus receptor activity	Upregulation
**MMP family**	Increase (Fang et al., 2022)	CVS-B2c-infected mouse brain	MMP9	GO:0030198-extracellular matrix organization	Upregulation

**Table 2:** Previously identified proteins or genes in RABV-infected *in vivo* tissue samples and genes expressed only in the RABV-infected 3D hiPSC-derived neuronal model.

Genes expressed only in infected 3D and not in infected 2D culture models					
Reported proteins/genes in RABV-infected *in vivo* samples			Related genes found in RABV-infected 3D hiPSC-derived neuronal model		
Protein/gene	Expression change (reference)	Sample	Gene	GO: ID-Term name	Expression change
**Nitrate** **Nitric oxide** **iNOS**	Increase (Madhu et al., 2016) Increase (Hooper et al., 1995)	CVS-infected mouse serum CVS-24-infected rat brain	NOS1 NOS2 NOS3	GO:0033555-multicellular organism response to stress GO:0003013-circulatory system process	Upregulation
**Catecholamines**	Increase (Fu and Jackson, 2005)	CVS-infected mouse brain	Tyrosine Hydroxylase	GO:0001505-regulation of neurotransmitter levels	Upregulation
**CXCL10**	Increase (Long et al., 2020)	GD-SH-01-infected mouse brain	CXCL10	GO:0002237-response to molecule of bacterial origin GO:0005576-extracellular region	Upregulation
**CX3CL1**	Increase (Appolinário et al., 2016)	Dog RABV and hematophagous bat RABV-infected mouse	CX3CL1	GO:0050896-response to stimulus GO0002376-immune system process	Upregulation
**UBE2N**	Increase (Mehta et al., 2016)	Street RABV (SRV) -infected mouse brain	UBE2N	GO:0019538-protein metabolic process GO:0006281-DNA repair	Upregulation
**TGFB family**	(Wang et al., 2005)	CVS-B2c-infected mouse	TGFBR2	GO:0032502-developmental process GO:0002376-immune system process	Down regulation
**PDZ**	(Javier and Rice, 2011)	CVS-11-infected SH-SY5Y cells and mouse	FRMPD4	GO:0043197-dendritic spine GO:0005856-cytoskeleton	Down regulation
**GRM2**	(Wang et al., 2018)	ERA strain- and GX/09 RABV- infected mouse	GRM1	GO:0098978-glutamatergic synapse GO:0004930-g-protein-coupled receptor activity	Down regulation

### 3.4 Neuronal responses to different RABV strains

**Figure 6:**
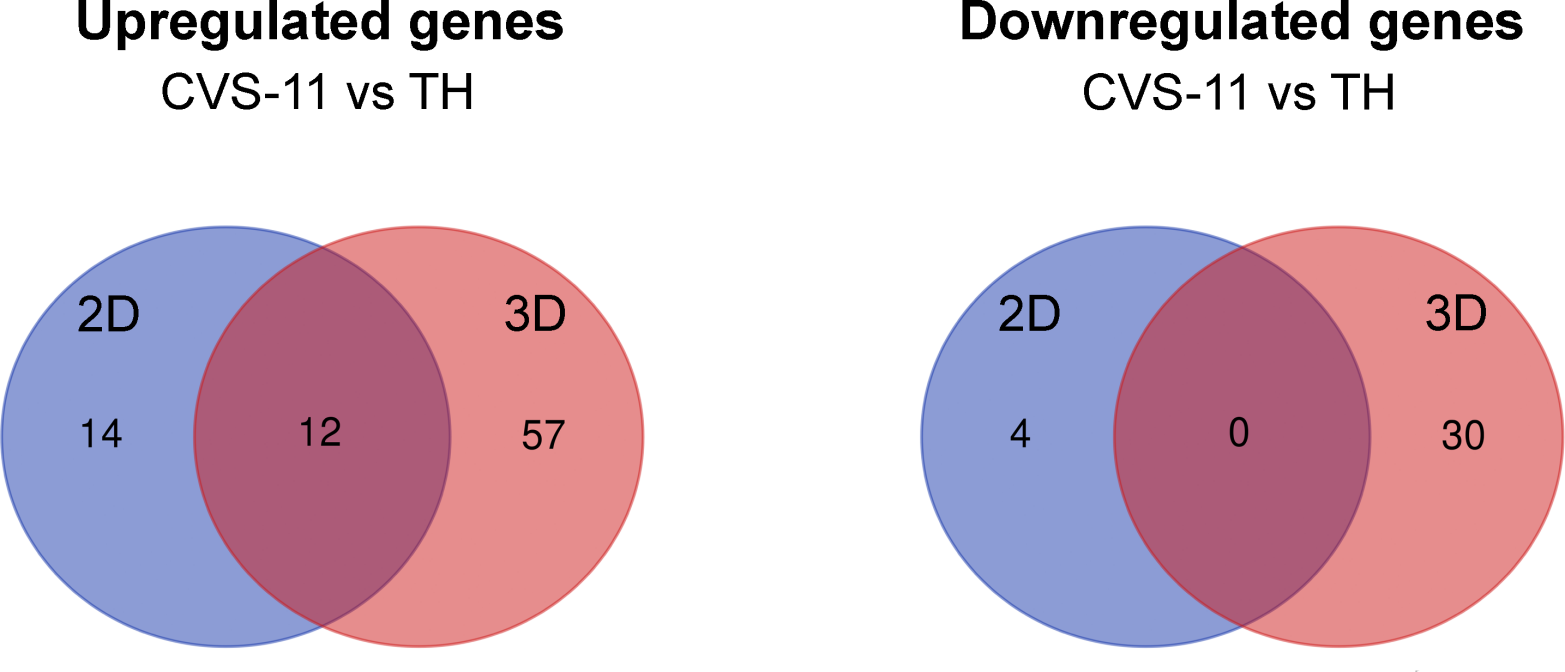
Differential gene expression from NanoString analysis of 2D and 3D hiPSC-derived neuronal models infected with CVS-11 or TH. HiPSC neurons seeded in 2D and 3D cultures were infected with CVS-11 or TH at an MOI of 0.5. At 24 hpi, cells were harvested for RNA isolation. The purified RNA was subjected to NanoString analysis and data normalization. Upregulated and downregulated genes were identified at a threshold of ≥ 1.5-fold change in expression levels. Venn diagrams showing the overlapping and different DEGs of CVS-11 compared to TH infected samples that were upregulated and downregulated across 2D and 3D models at 24 hpi.

To further evaluate gene expression induced by RABV strains, we asked whether there were genes either commonly or differentially expressed in response to different RABV strains. In the 3D model, there were 69 upregulated genes and 30 downregulated genes expressed in CVS-11-infected neurons compared to TH-infected neurons at 24 hpi (**Figure 6**). However, in the 2D model, 26 genes were upregulated and 4 genes were downregulated in CVS-11-infected neurons (**Figure 6**). Interestingly, 12 common upregulated genes were observed for the CVS-11- and TH-infected samples across both 2D and 3D models (**Figure 6**). These included genes associated with the galactosylceramide biosynthetic/metabolic process, galactolipid biosynthetic/metabolic process (GAL3ST1, FA2H), and dopamine metabolic process (DBH, PNKD) (**Supplementary File 5**). The 3D model yielded 57 more genes that were not seen in the 2D model (**Figure 6** and **Supplementary File 4**). These are involved in cytokine-cytokine receptor interactions (LIF, CSF1R, CXCL10, CCR5, CXCL3, IL4R, TNFRSF1A, NGFR), immune signaling pathways, signal transduction, apoptosis (TNFRSF1A, NGFR, HTRA2) and nitric oxide synthesis (NOS) (**Supplementary File 5**). Importantly, genes associated with collagen-containing ECM such as MMP2, ICAM1, EGFL7, MMRN2, and MMP9 were observed only in 3D conditions, suggesting a distinct characteristic of neurons embedded in the collagen-Matrigel in secreting essential substances required for cellular functions. Thirty of the genes downregulated in CVS-11-infected samples compared with TH-infected samples observed only in the 3D model (**Figure 6** and **Supplementary File 6**) were associated with pre- and post-synaptic compartments modulating chemical synaptic transmissions such as GRIN2A, GRM2, ADCY8, PRKCG, and PTEN.

### 3.5 GO and KEGG analyses of genes expressed in RABV-infected 3D neuronal culture

We next performed functional enrichment analysis using the STRING database to examine the relationship of DEGs observed in RABV-infected neurons in the 3D model at 24 hpi. Upregulated genes from CVS-11-infected compared to TH-infected 3D samples were clustered into 6 groups based on a kmeans clustering (**Supplementary Figure 5**). GO and KEGG analyses (**Supplementary Figure 6** and **Supplementary File 5**) revealed that the genes are involved in cytokine-mediated signaling pathways, neurotransmitter metabolic processes, regulation of synaptic transmission (dopaminergic), regulation of cell population proliferation, and innate immune responses. These include several important genes known for mediating inflammatory responses such as NOS1, ICAM1, CXCL10, CX3CL1, and HMOX1. Changes in neuronal function-associated genes were also observed such as DBH, NOS1, Tyrosine Hydroxylase, DRD4 (regulation of neurotransmitter levels), and DRD1, NTRK1 (regulation of trans-synaptic signaling). Several DEGs related to divalent inorganic cation homeostasis were also observed in our model, including ITPR3, NOS1, CD40, EPO, SLC11A1 and CXCL10. These genes are essential for maintaining intracellular calcium ion levels, which is important for cell survival, neuronal function, and response to virus infection (Sui et al., 2006). Upregulation of genes and proteins regulating calcium homeostasis has previously been observed during early RABV infection (Kanu et al., 2021).

Downregulated genes were likewise enriched and clustered using the STRING database (**Supplementary Figure 7**). GO and KEGG analyses revealed that these genes were involved in the regulation of postsynaptic membrane potential (GRM1, GRIN2A, and BDNF), cellular component organization (ADCY8, INSR, NLRP3, NR4A2, HDAC1, FRMPD4, TF, PLA2G4C), glutamatergic synapse function (PRKCG, ADCY8, GRM1, ITPR2, GRM2, GRIN2A, and PLA2G4C) and GABAergic synapse function (PRKCG, ADCY8, CACNA1D) (**Supplementary Figure 8** and **Supplementary File 6**). Such downregulation of genes associated with synaptic/trans-synaptic transmission in CVS-11 infection may possibly result in a greater degree of neuronal dysfunction compared to TH infection.

### 3.6 Validation of NanoString gene transcripts

**Figure 7:**
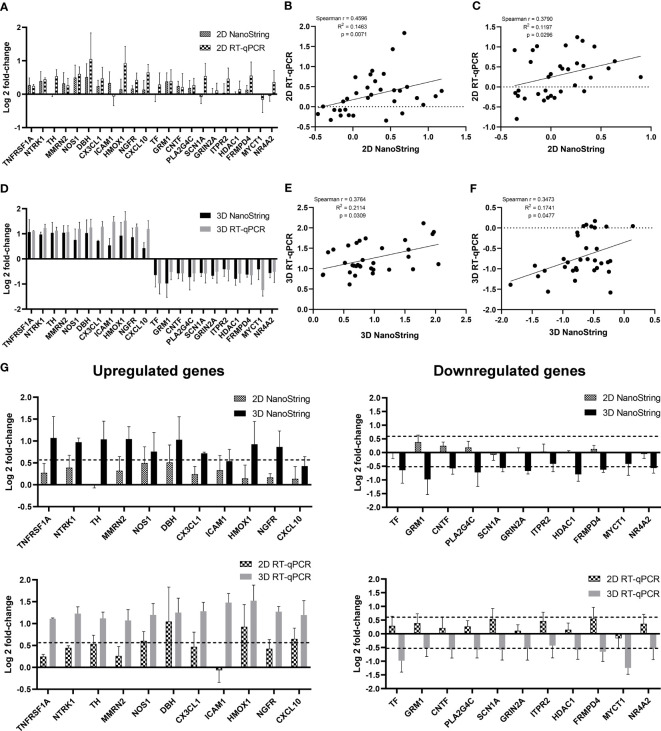
Comparison of DEG expression levels as measured by NanoString and RT-qPCR. HiPSC neurons seeded in 2D and 3D cultures were infected with CVS-11 or TH at an MOI of 0.5. At 24 hpi, cells were harvested for RNA isolation. The purified RNA was subjected to NanoString analysis and data normalization. Upregulated and downregulated genes were identified at a threshold of ≥ 1.5-fold change in expression levels. **(A)** Log_2_-fold change of expression levels and **(B)** Spearman correlation scattered plot of selected upregulated genes and **(C)** downregulated genes from CVS-11 infected samples relative to the TH infected samples in 2D culture as measured by NanoString and RT-qPCR. Each data point of the scattered plot represents a raw value from three independent experimental batches. **(D)** Log_2_ fold-change of expression values and **(E)** Spearman correlation scattered plot of selected upregulated genes and **(F)** downregulated genes from CVS-11-infected samples relative to the TH-infected samples in 3D culture as measured by NanoString and RT-qPCR. Each data point of the scattered plot represents a raw value from three independent experimental batches. **(G)** Comparison of log_2_-fold change of upregulated and downregulated genes from 2D and 3D models by NanoString and RT-qPCR analysis. Dotted lines represent the cut-off threshold of 1.5-fold change (log_2_1.5 ≈ 0.58 for upregulated genes and log_2_(0.7) ≈ -0.51 for downregulated genes). Data are presented as mean ± SEM, *n* = 3.

Based on the functional enrichment of upregulated and downregulated genes, we have selected 11 upregulated and 11 downregulated genes as representatives for validation via RT-qPCR and functional assays. Count numbers obtained from NanoString analysis were compared between infected samples to examine the dynamics of gene expression for each gene (**Supplementary Figure 9**). Positive correlations (*p* value < 0.05) were observed between Log_2_ fold-changes in the expression values from NanoString and RT-qPCR for both the 2D model (**Figures 7A–C**) and the 3D model (**Figures 7D–F**). In addition, the differences in DEG levels between 2D and 3D models as detected by RT-qPCR corresponded with NanoString data (**Figure 7G**).

### 3.7 CVS-11 grown in 3D culture significantly enhanced NOS production

**Figure 8:**
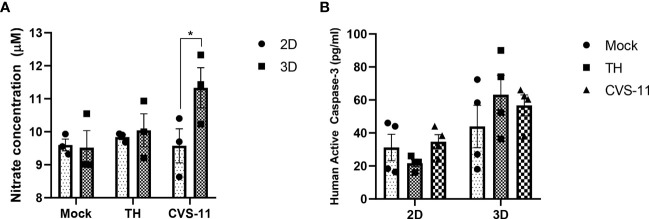
Nitric oxide and caspase-3 production in 2D and 3D neuronal culture models infected with different strains of RABV. HiPSC neuronal cells were seeded in 2D and 3D cultures and infected with CVS-11 or TH at an MOI of 0.5. At 24 hpi, **(A)** culture supernatants were collected and assessed for nitrite concentrations by a nitric oxide production assay, and **(B)** cells were harvested, lysed, and assessed for active caspase-3 levels using a caspase-3 ELISA assay. Data are presented as mean ± SEM, *n* = 3 for a NOS assay and *n* = 4 for a caspase-3 ELISA assay. *p<0.05.

Nitric oxide synthase (NOS) was one of the genes uniquely expressed in infected 3D cultures only and has been reported to be induced by RABV infection in brain samples (Madhu et al., 2016). CVS-24-infected rat brains showed enhanced NOS enzymatic activity at 3 days post-infection (Hooper et al., 1995). As a result, we selected NOS to evaluate an aspect of neuronal responses to RABV infection using a nitric oxide production assay. The results showed that nitrate concentrations in the supernatant of CVS-11-infected neurons were significantly higher than those of TH-infected neurons in the 3D model despite no differences being detected in the 2D model (**Figure 8A**). As nitric oxide production is associated with the intrinsic apoptotic pathway (Wei et al., 2000), we further examined whether an elevation in nitrate levels would lead to apoptotic cell death following RABV infection using Caspase-3 (a key effector enzyme in inducing cell apoptosis) ELISA assay. We found there was no significant difference in active caspase-3 in CVS-11- and TH-infected samples in both 2D and 3D models (**Figure 8B**). Taken together, these data suggest that CVS-11-infected neurons in 3D model significantly enhanced NOS production, which was not seen in 2D model, and this did not result in Caspase-3-mediated cell apoptosis.

## 4 Discussion

### 4.1 Matrigel improved survival and neuronal maturation of hiPSC-derived neurons in collagen hydrogels

Collagen hydrogels have been widely used as scaffolds to support neurogenesis as well as to fabricate neuronal models since it is one of the main components of ECM present in the native nervous tissues (Alovskaya et al., 2007). A previous study, however, showed that collagen type I hydrogels were detrimental to human neural stem cells (hNSCs) whereas the addition of Matrigel improved cell viability, proliferation, and differentiation of the hNSC neuronal network (Vagaska et al., 2020). This corresponds to our study results, where collagen-Matrigel blends enhanced cell viability and metabolic activity compared to pure collagen hydrogels. In addition, collagen-Matrigel blends provided a favorable environment for neuronal network formation, maturation, and differentiation as indicated by a more complex neuronal network and robust induction of mature neuronal (MAP2) and synaptic markers (PSD95, Synapsin1). The presence of Matrigel in collagen hydrogel blends greatly promoted cell survival of hiPSC-derived neurons likely by providing a variety of integrins including laminin to support neuron growth (Wang et al., 2020). Our data demonstrated the potential of fabricated 3D collagen-Matrigel scaffolds for the study of viral infection to closely mimic the CNS. These hydrogel-based 3D neuronal models provide the native-like ECM microenvironment, cell-matrix interactions, and homogenous cellular compositions which are limited in organoids.

### 4.2 Distinct virus behaviors detected in a 3D neuronal culture model

Our study showed that different RABV strains grow differently in 3D but not 2D culture. TH (wild-type Thai canine rabies isolate) showed significantly greater growth kinetics than CVS-11 (laboratory reference strain) in 3D culture. This indicates that different culture systems of the same host cells may affect virus behaviors, as previously reported for influenza virus (Berg et al., 2018) and hepatitis C virus (HCV) (Molina-Jimenez et al., 2012), where 3D cell cultures provided more physiological infection patterns.

Interactions between pathogens and ECM such as collagen, laminin, fibronectin, and proteoglycans have been shown to play an essential role in pathogenesis as well as immune responses to infection (Singh et al., 2012; Tomlin and Piccinini, 2018). The 3D collagen environment can reduce diffusion rates of human immunodeficiency virus type 1 (HIV-1) and impede virus production (Imle et al., 2019). In addition, HIV-1 proteins gp120 and gp160 can bind to the C-terminal heparin-binding domain of circulating fibronectin and laminin which could help clear virions from the bloodstream (Bozzini et al., 1998). Human papillomavirus (HPV) uses heparan sulfate as the primary attachment receptor and can strongly bind laminin 5, mediating its entry into host cells (Richards et al., 2014; DiGiuseppe et al., 2017). Heparan sulfate also serves as an attachment receptor and supports CVS infection in SK-N-SH cells (Sasaki et al., 2018). Shuai et al. (2020) found that the RABV strain ERA uses integrin β-1, transmembrane cell surface molecules, as the peripheral entry target (Shuai et al., 2020). It is noted that integrins are known to mediate cell-cell and cell-ECM signaling pathways. These highlight the potential value of 3D cultures for modeling host-RABV interactions.

### 4.3 Changes in genes related to inflammatory and immune response in CVS-11-infected 3D neuronal culture model

Our results showed that a group of inflammatory and immune response-related genes, particularly in the cytokine-cytokine receptor interaction pathway, was strongly upregulated in CVS-11 infected 3D model at 24 hpi when compared to TH. These include CX3CL1, NGFR, LIF, CXCL10, TNFRSF12A and ICAM1. CX3CL1 is a chemokine that mediates communication between neurons and microglia, also known as CX3CL1-CX3CR1 axis. This signaling regulates inflammatory response and maintains homeostasis in the brain upon infection. Although CX3CL1 exerts anti-inflammatory role in the induction of infiltrating inflammatory cells such as monocytes and leukocytes as seen in herpes simplex virus infection (Menasria et al., 2017), a high expression of this gene is detrimental to hippocampal damage during Theilor virus infection (Käufer et al., 2018). RABV-ERA strain infection in mouse brain at day 7 showed many differentially expressed upregulated miRNAs involved in immune-related pathway including mmu-miR-183 that potentially regulates CX3CL1 (Zhao et al., 2012). Additionally, several previous studies reported an increase in the expression level of CXCL10 in RABV-infected human mouse brain (Zhang et al., 2016; Feige et al., 2021). CXCL10 belongs to proinflammatory chemokines that mainly functions in modulate trafficking of T cells to the CNS to promote viral clearance. CXCL10 expression was highly detected in neurons and microglia over 9 days of infection with both CVS-B2c and DRV-Mexico in mouse brain suggesting the neuro-glia communication response (Chai et al., 2014). CVS-11 has also been demonstrated to increase CXCL10 expression in several animal model aligning with the result observed in our 3D neuronal model (Zhao et al., 2009; Zhang et al., 2016). Furthermore, MMP9, which produces proteins of the matrix metalloproteinase family involved in ECM degradation in physiological processes, was highly upregulated in CVS-11 infected 3D model as compared to TH. MMPs are known as inflammatory mediators that facilitate remodeling and recruitment of cells into the infected tissue (Chen et al., 2013). MMP8 and MMP9 were upregulated in the mouse brain infected with CVS-B2c at 6 days post-infection which helped in enhancing blood-brain barrier (BBB) permeability (Fang et al., 2022).

### 4.4 CVS-11-infected 3D neuronal cells revealed significant NOS production and neuronal dysfunction

In our study, CVS-11 induced greater amounts of NOS expression and higher nitrate concentrations compared to TH in the 3D model. Nitric oxide (NO), a free radical gas whose production is catalyzed by nitric oxide synthase (NOS), plays an essential role in transmitting signals in organisms. In the CNS, neuronal NOS (NOS1) is responsible for the synthesis of NO by neurons and mediates neurotransmission and neurotoxicity. During virus infection, intracellular NO production increases particularly by inducible NOS, to facilitate host responses (Lisi et al., 2021). Previous studies reported increased nitrate concentrations in CVS-infected mice serum from day 2–12 post-infection (Madhu et al., 2016). High NOS2 mRNA levels and NOS enzymatic activity have been detected in rat brains infected with the CVS-24 strain of RABV 3 days after infection along with the onset of clinical signs of disease (Hooper et al., 1995). It has been also reported that NO can mediate microglia phagocytosis which may help in the inhibition of virus replication (Maksoud et al., 2019). We postulate that higher amounts of nitrate in CVS-11-infected neurons might be related to virus neuropathogenesis, contributing to the lower replication kinetics observed in the CVS-11 strain in the 3D model. The antiviral effect of NO may mitigate the replication of viruses as shown in previous studies on RABV and other viruses (Ubol et al., 2001; Xue et al., 2019; Akaberi et al., 2020). On the other hand, NO accumulation in the brain may exert a neurotoxic effect leading to neuronal dysfunction and altered brain function (Koprowski et al., 1993; Van Dam et al., 1995). This may be reflected in the downregulated genes involved in the regulation of synaptic transmission as seen in the CVS-11-infected 3D neuronal model.

Neuronal dysfunction is one of the common hallmarks of RABV. Here, we demonstrated altered expression of several genes following CVS-11 infection in the 3D model which are involved in the regulation of neurotransmitter levels and the regulation of trans-synaptic signaling. These genes include DBH, DRD1, DRD4, and Tyrosine Hydroxylase, which mainly function in the release of dopamine, which belongs to the catecholamine neurotransmitter group. A previous clinical study reported defective dopamine and serotonin biosynthesis in the cerebrospinal fluid (CSF) of RABV-infected patients (Willoughby et al., 2009). Impaired neurotransmission of serotonin and other catecholamines from the brainstem of street RABV-infected skunks has been shown previously (Charlton et al., 1984). In the case of downregulated genes, several genes are involved in glutamate receptor activity including GRM1, GRM2, GRIN2A, and glutamatergic synapses such as PRKCG, ADCY8, GRM1, and ITPR2. A recent study showed direct interaction between the glutamate metabotropic receptor (GRM2) and the G protein of the RABV strain ERA, facilitating virus entry *in vitro* (Wang et al., 2018). The PRKCG gene encodes protein kinase C (PKC) which is essentially expressed in the brain and spinal cord and is involved in neuronal long-term synaptic potentiation. PKC subunits have been previously shown to influence the phosphorylation of the RABV P protein (Besson et al., 2019), suggesting the significance of this mechanism for RABV replication. Many of the genes related to synaptic activity were uniquely downregulated in CVS-11 infection as compared with TH which might potentially result in the impaired functioning of neuronal processes.

Comparatively, CVS-11 infection in a 3D neuronal model exhibited more pronounced enhancements in inflammatory and immune responses, as well as neuronal dysfunction, in contrast to TH. This discrepancy might be attributed to variances in virus replication, pathogenesis and the impact of 3D microenvironment, highlighting the significance of using 3D model for studying virus tropism. Nevertheless, a conclusive assessment of the neurovirulence and neuroinvasiveness of these two strains necessitates further investigation through an *in vivo* study.

Although our 3D model has provided a panel of gene responses following RABV infection, it still does not mimic the complexity of the *in vivo* CNS regarding the different cell types and the formation of cortical tissue structures. The 3D co-culture model containing astrocytes or microglia will better recapitulate the *in vivo* situation and allow us to gain deeper insights into the relationship between host cells and virus tropism. Furthermore, comprehending the impact of viruses on cytoarchitecture and neuronal electrophysiology may necessitate long-term culture techniques, modifications to the hydrogel, or the utilization of an organoid platform to construct a fully functional and mature cortical-like 3D model (Su et al., 2022). Whether the unique gene expression profiles in 2D and 3D model contribute to the differences in RABV growth kinetics observed in our *in vitro* model remains to be explored. In addition, it would be worthwhile to compare virus growth kinetics and pathogenesis in animal models to those in the 3D model. Virus behaviors can also be influenced by various factors such as MOI and host cell density and conducting more investigations would certainly strengthen knowledge of RABV infection in the CNS.

In summary, we established a 3D hydrogel-based neuronal model using hiPSC-derived neurons to study RABV infection. HiPSC-derived neurons in our 3D model differ significantly from the conventional 2D monolayer model in terms of cellular architecture, morphology, neuronal differentiation, maturation and long-term stability, allowing investigation into more *in vivo*-like host-virus interactions. Two different RABV strains (TH and CVS-11) exhibited more distinct growth kinetics and increased DEGs in the 3D neuronal model as compared to the 2D model, implying an impact of the surrounding matrix environment on host cell-virus interactions. These results highlight the potential use of the 3D hydrogel-based hiPSC-derived neuronal model for RABV-host interactions to closely mimic *in vivo* pathology and a remarkable opportunity for new therapeutics and vaccines against rabies.

## Data availability statement

The data presented in the study are deposited in the GEO repository, accession number GSE231664.

## Ethics statement

The use of hiPSCs was approved by the Institutional Review Board (IRB) Ethics Committee of the National Science and Technology Development Agency (NSTDA) under the project title “Biological and molecular study on Thai street rabies virus infection in human induced pluripotent stem cells (hiPSC) derived neurons” (Approval number: NIRB-038-2562, Date of approval: 14th January 2021).

## Author contributions

PM performed 2D and 3D cultures and characterization, RABV growth curve analysis, and RNA sample preparation, analyzed the data, and drafted the manuscript. PM and TC performed NPC and hiPSCs-derived neuronal cultures. NT prepared RABV stocks and performed RABV infection and titration. RW carried out confocal microscopy. CK conceptualized the study, provided resources, analyzed the data, and drafted, reviewed, and edited the final manuscript. PL provided resources. PL and CK participated in funding acquisition. All authors contributed to the article and approved the submitted version.

## Supplementary material

Supplementary Figure 1 Characterization of the pre-differentiated neurons. 10-day hiPSC-derived neurons were seeded in 2D monolayer and stained for **(A)** cortical layer specific markers CTIP2 (green), βIII-tubulin (red) and for **(B)** postsynaptic density protein 95, PSD95 (red), Phalloidin (green). Cells were imaged using fluorescence microscopy after 24 h. Scale bars = 20 µm

Supplementary Figure 2 The positive control linearity QC. Number of counts for external RNA control consortium (ERCC) consist of 6 synthetic DNA positive control plotted against the different concentrations tested (0.125–128 fM). The correlation coefficients (R^2^) of a linear fit to the mean of positive counts in each group (n = 14) are indicated for the entire concentration range tested.

Supplementary Figure 3 Phase contrast images of 2D and 3D cultures at 2 h and 168 h. HiPSC-derived neurons were seeded in 2D monolayer and 3D 1:1.25 mg/ml collagen-Matrigel hydrogel blends at the density of 1×10^6^ cells/ml. Cells were imaged using bright field microscopy at specified time points. HiPSC-derived neurons tended to aggregate in 2D culture but not in 3D culture when cultured over a longer period. Scale bars = 50 µm; COL, Collagen; MAT, Matrigel.

Supplementary Figure 4 Heat maps showing changes of gene expressions following RABV-TH and CVS-11 infection in 2D and 3D culture models. HiPSC neurons seeded in 2D and 3D cultures were infected with CVS-11 or TH at an MOI of 0.5. At 8 hpi and 24 hpi, cells were harvested for RNA isolation. The purified RNA was subjected to NanoString analysis and data normalization. The expression levels are indicated in log 2-fold-change over mock.

Supplementary Figure 5 Functional enrichment of upregulated genes in the CVS-11 infected neurons in the 3D model using STRING database. The genes were clustered in to 6 groups (as presented in different colors) using kmean clustering method. The edges represented in the full network indicate both functional and physical protein associations. Detail of each cluster and protein description was shown in **Supplementary file 2**. Network statistics - number of nodes: 69, number of edges: 150, PPI enrichment p-value: <1.0×10^-16^.

Supplementary Figure 6 Pathway analysis of upregulated genes in the CVS-11 infected neurons (compared with TH) in the 3D model. **(A)** GO Biological Process terms and **(B)** KEGG pathways of upregulated genes were analyzed using ShinyGO 0.77 based on Ensembl database (http://bioinformatics.sdstate.edu/go/).

Supplementary Figure 7 Functional enrichment of downregulated genes in the CVS-11 infected neurons in the 3D model using STRING database. The genes were clustered in to 5 groups (as presented in different colors) using kmean clustering method. The edges represented in the full network indicate both functional and physical protein associations. Detail of each cluster and protein description was shown in **Supplementary file 2**. Network statistics - number of nodes: 30, number of edges: 59, PPI enrichment p-value: <3.07×10^-12^.

Supplementary Figure 8 Pathway analysis of downregulated genes in the CVS-11 infected neurons (compared with TH) in the 3D model. **(A)** GO Biological Process terms and **(B)** KEGG pathways of downregulated genes were analyzed using ShinyGO 0.77 based on Ensembl database (http://bioinformatics.sdstate.edu/go/).

Supplementary Figure 9 NanoString normalized count data of differentially expressed genes. Samples were infected with RABV-TH and RABV-CVS-11 at MOI of 0.5 for 24 h. Total RNA was collected for both 2D and 3D culture samples at 0, 8, and 24 hpi via TRIzol reagent. One hundred nanograms of the purified total RNA were analyzed using the nCounter Analysis System. Normalized count data of selected 22 differentially expressed genes included **(A)** 11 upregulated genes and **(B)** 11 downregulated genes.

Supplementary File 1 Fold-changes of all genes in 2D and 3D models.

Supplementary File 2 Upregulated DEGs in 2D and 3D models.

Supplementary File 3 Downregulated DEGs in 2D and 3D models.

Supplementary File 4 Distinct and overlapping DEGs across virus strains and culture models.

Supplementary File 5 GO and KEGG analyses of upregulated DEGs.

Supplementary File 6 GO and KEGG analyses of downregulated DEGs.
