# Supplementary figures and images for: Hydrogel-based 3D human iPSC-derived neuronal culture for the study of rabies virus infection
Source: Front Cell Infect Microbiol. 2023 Aug 25;13:1215205. doi: 10.3389/fcimb.2023.1215205 (PMC10485840; doi:10.3389/fcimb.2023.1215205)

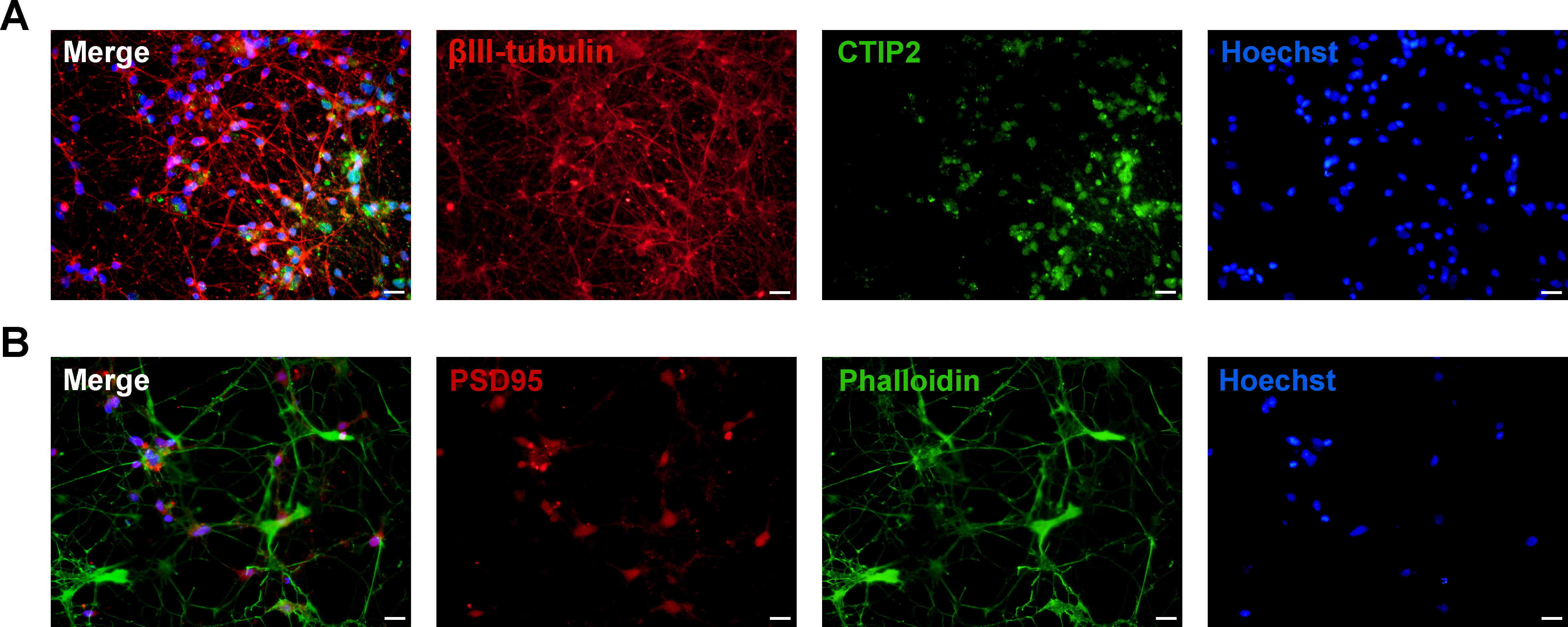

Supplement: Supplementary Figure 1 — Characterization of the pre-differentiated neurons. 10-day hiPSC-derived neurons were seeded in 2D monolayer and stained for (A) cortical layer specific markers CTIP2 (green), βIII-tubulin (red) and for (B) postsynaptic density protein 95, PSD95 (red), Phalloidin (green). Cells were imaged using fluorescence microscopy after 24 h. Scale bars = 20 µm [file Image_1.tif]

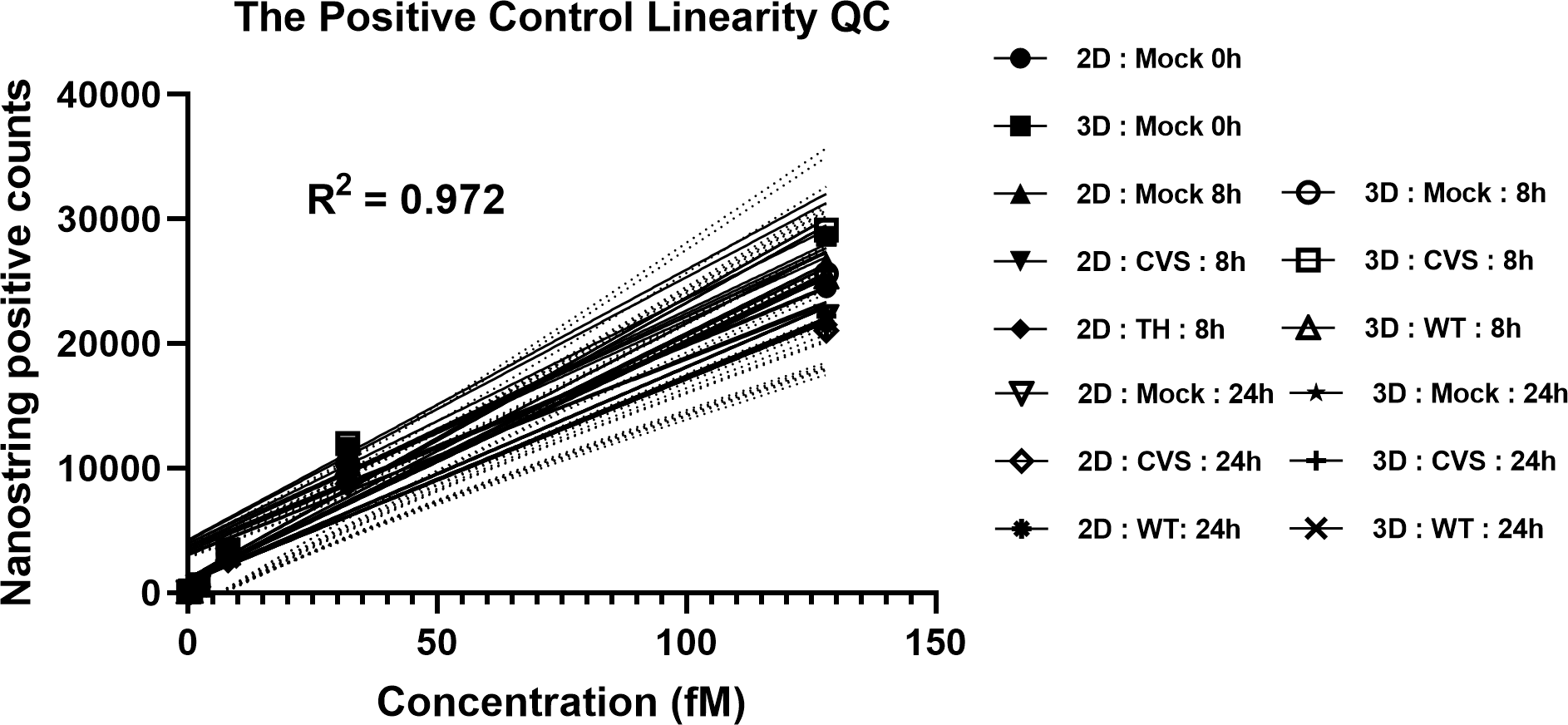

Supplement: Supplementary Figure 2 — The positive control linearity QC. Number of counts for external RNA control consortium (ERCC) consist of 6 synthetic DNA positive control plotted against the different concentrations tested (0.125–128 fM). The correlation coefficients (R2) of a linear fit to the mean of positive counts in each group (n = 14) are indicated for the entire concentration range tested. [file Image_2.tif]

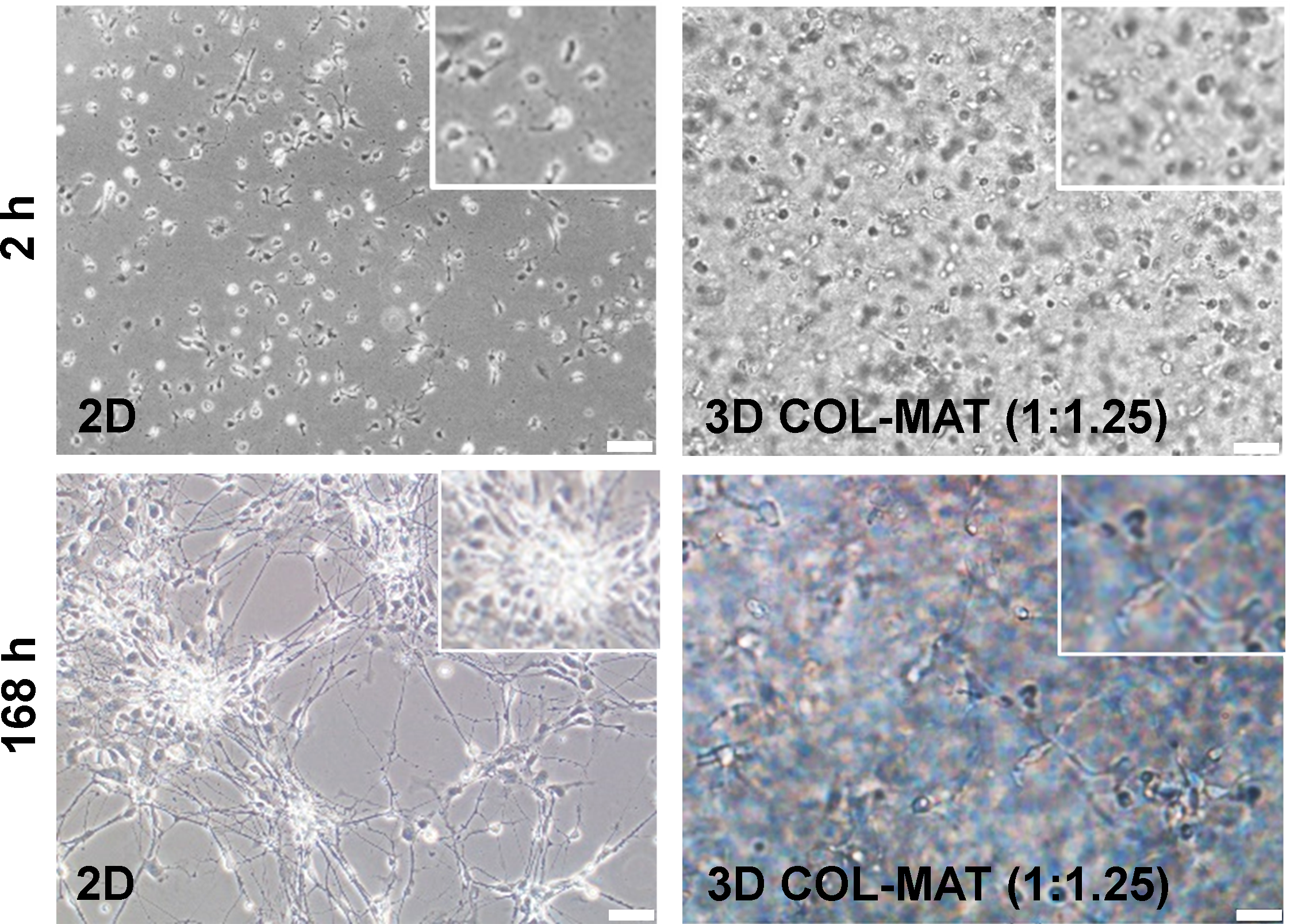

Supplement: Supplementary Figure 3 — Phase contrast images of 2D and 3D cultures at 2 h and 168 h. HiPSC-derived neurons were seeded in 2D monolayer and 3D 1:1.25 mg/ml collagen-Matrigel hydrogel blends at the density of 1×10^6^ cells/ml. Cells were imaged using bright field microscopy at specified time points. HiPSC-derived neurons tended to aggregate in 2D culture but not in 3D culture when cultured over a longer period. Scale bars = 50 µm; COL, Collagen; MAT, Matrigel. [file Image_3.tif]

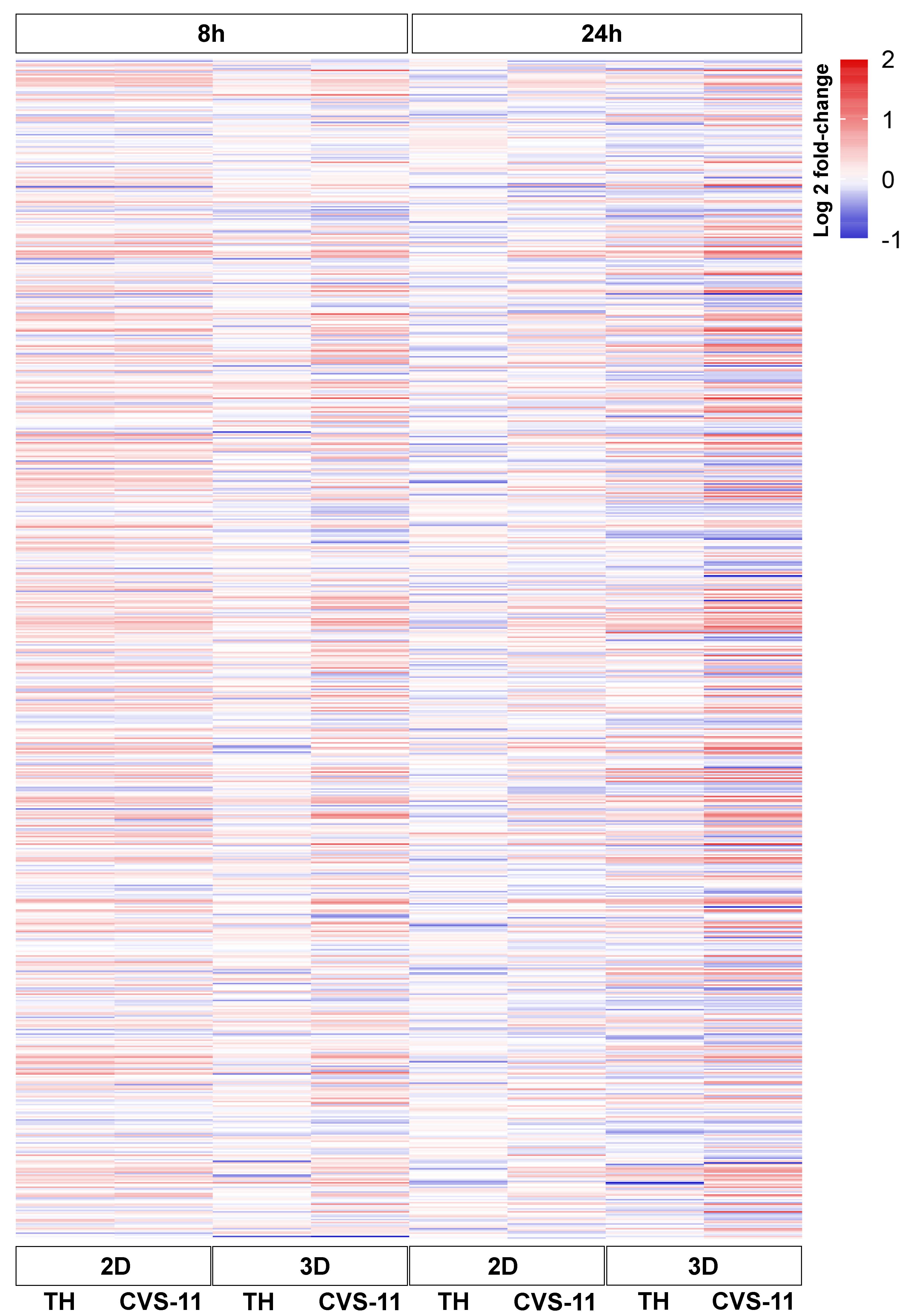

Supplement: Supplementary Figure 4 — Heat maps showing changes of gene expressions following RABV-TH and CVS-11 infection in 2D and 3D culture models. HiPSC neurons seeded in 2D and 3D cultures were infected with CVS-11 or TH at an MOI of 0.5. At 8 hpi and 24 hpi, cells were harvested for RNA isolation. The purified RNA was subjected to NanoString analysis and data normalization. The expression levels are indicated in log 2-fold-change over mock. [file Image_4.tif]

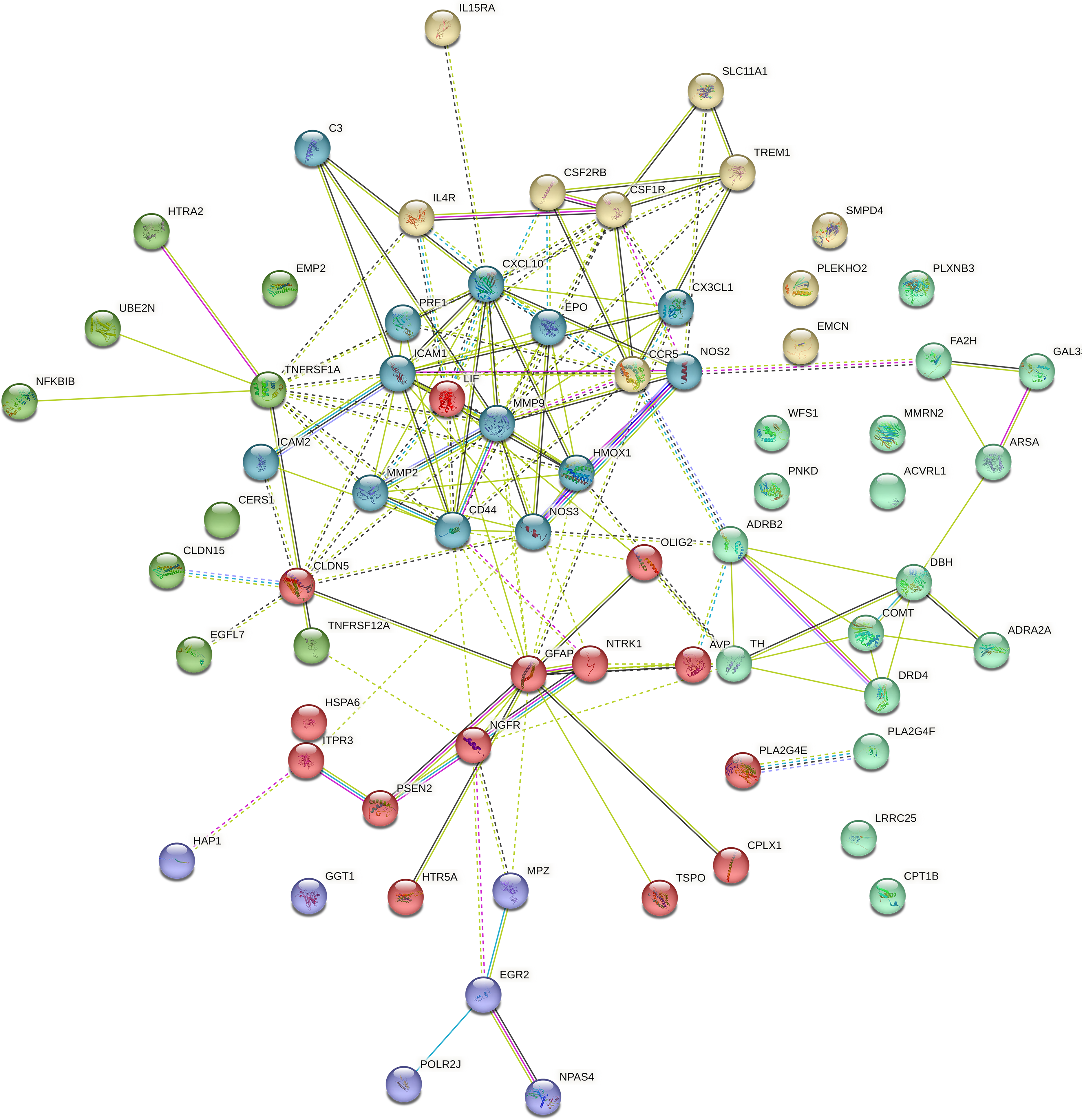

Supplement: Supplementary Figure 5 — Functional enrichment of upregulated genes in the CVS-11 infected neurons in the 3D model using STRING database. The genes were clustered in to 6 groups (as presented in different colors) using kmean clustering method. The edges represented in the full network indicate both functional and physical protein associations. Detail of each cluster and protein description was shown in Supplementary file 2 . Network statistics - number of nodes: 69, number of edges: 150, PPI enrichment p-value: <1.0×10^-16^. [file Image_5.png]

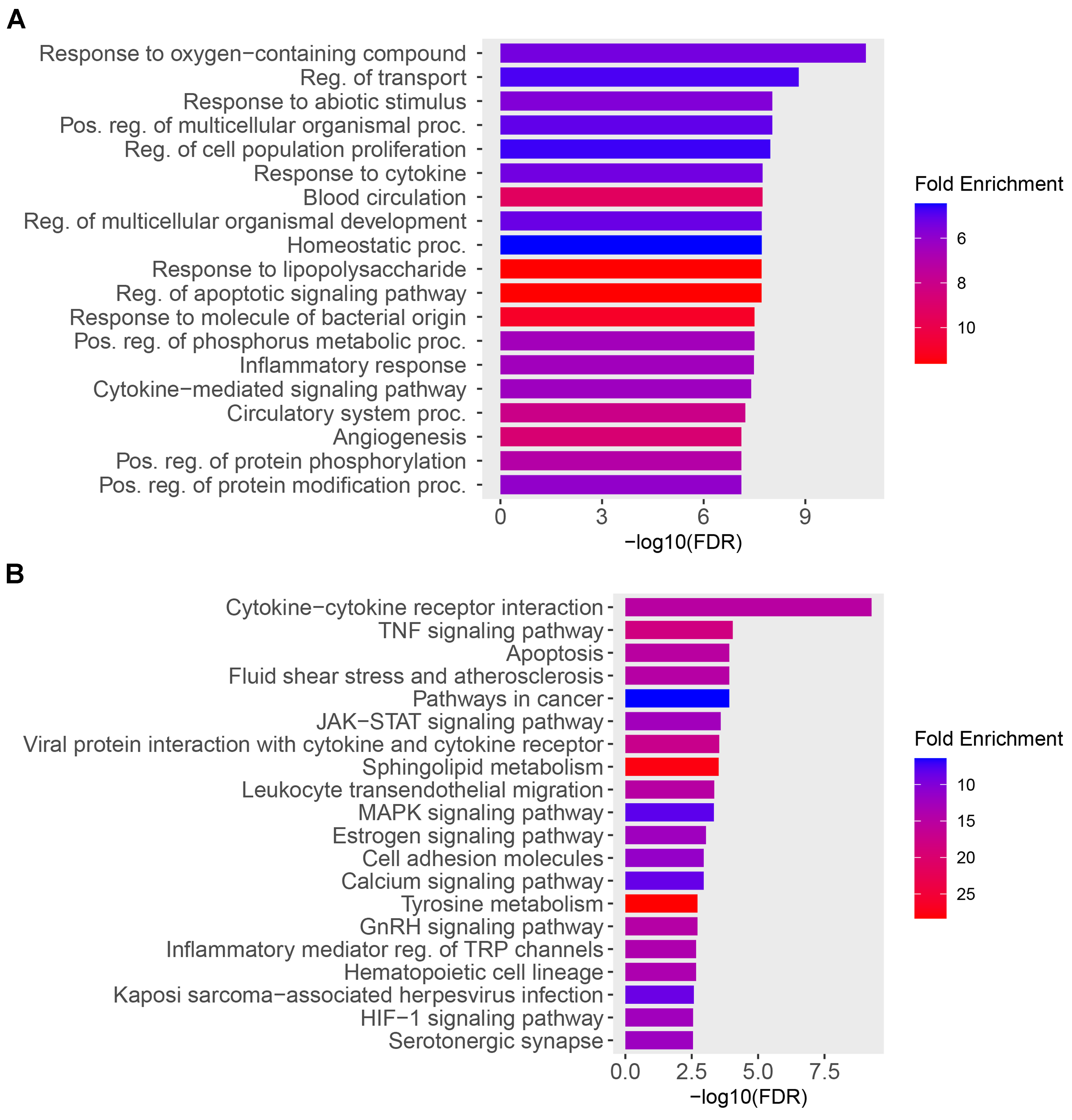

Supplement: Supplementary Figure 6 — Pathway analysis of upregulated genes in the CVS-11 infected neurons (compared with TH) in the 3D model. (A) GO Biological Process terms and (B) KEGG pathways of upregulated genes were analyzed using ShinyGO 0.77 based on Ensembl database (http://bioinformatics.sdstate.edu/go/). [file Image_6.tif]

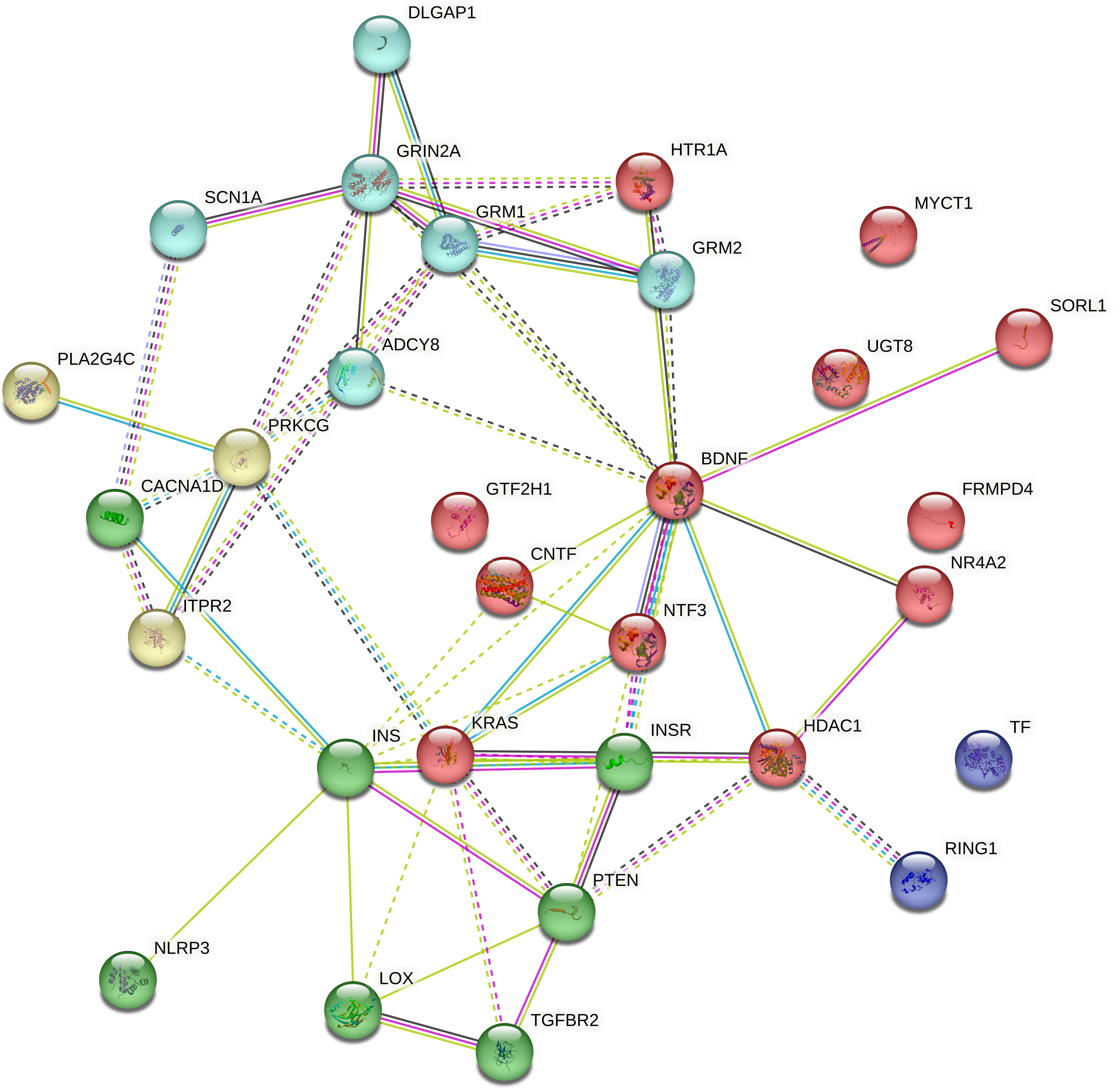

Supplement: Supplementary Figure 7 — Functional enrichment of downregulated genes in the CVS-11 infected neurons in the 3D model using STRING database. The genes were clustered in to 5 groups (as presented in different colors) using kmean clustering method. The edges represented in the full network indicate both functional and physical protein associations. Detail of each cluster and protein description was shown in Supplementary file 2 . Network statistics - number of nodes: 30, number of edges: 59, PPI enrichment p-value: <3.07×10^-12^. [file Image_7.tif]

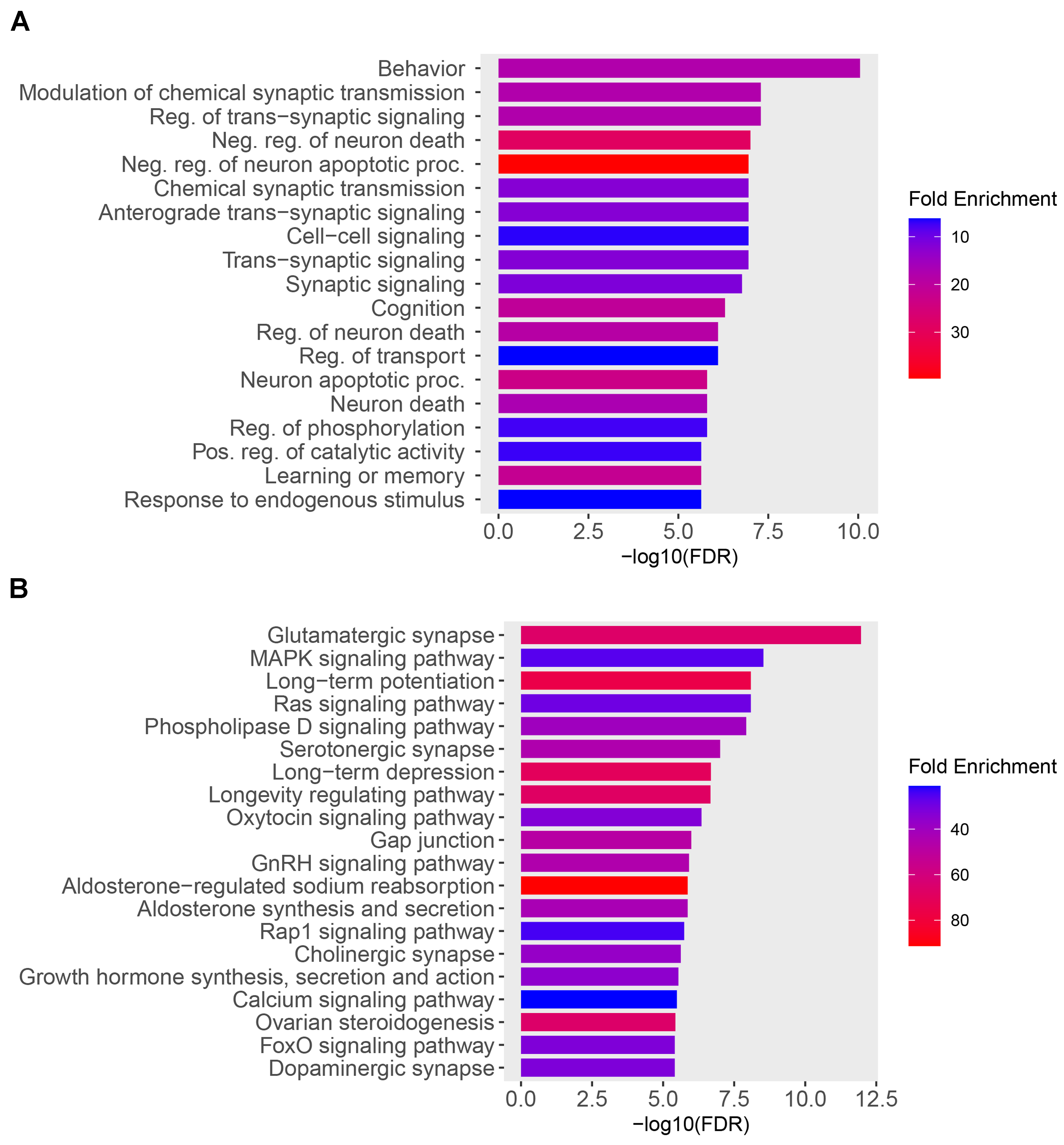

Supplement: Supplementary Figure 8 — Pathway analysis of downregulated genes in the CVS-11 infected neurons (compared with TH) in the 3D model. (A) GO Biological Process terms and (B) KEGG pathways of downregulated genes were analyzed using ShinyGO 0.77 based on Ensembl database (http://bioinformatics.sdstate.edu/go/). [file Image_8.tif]

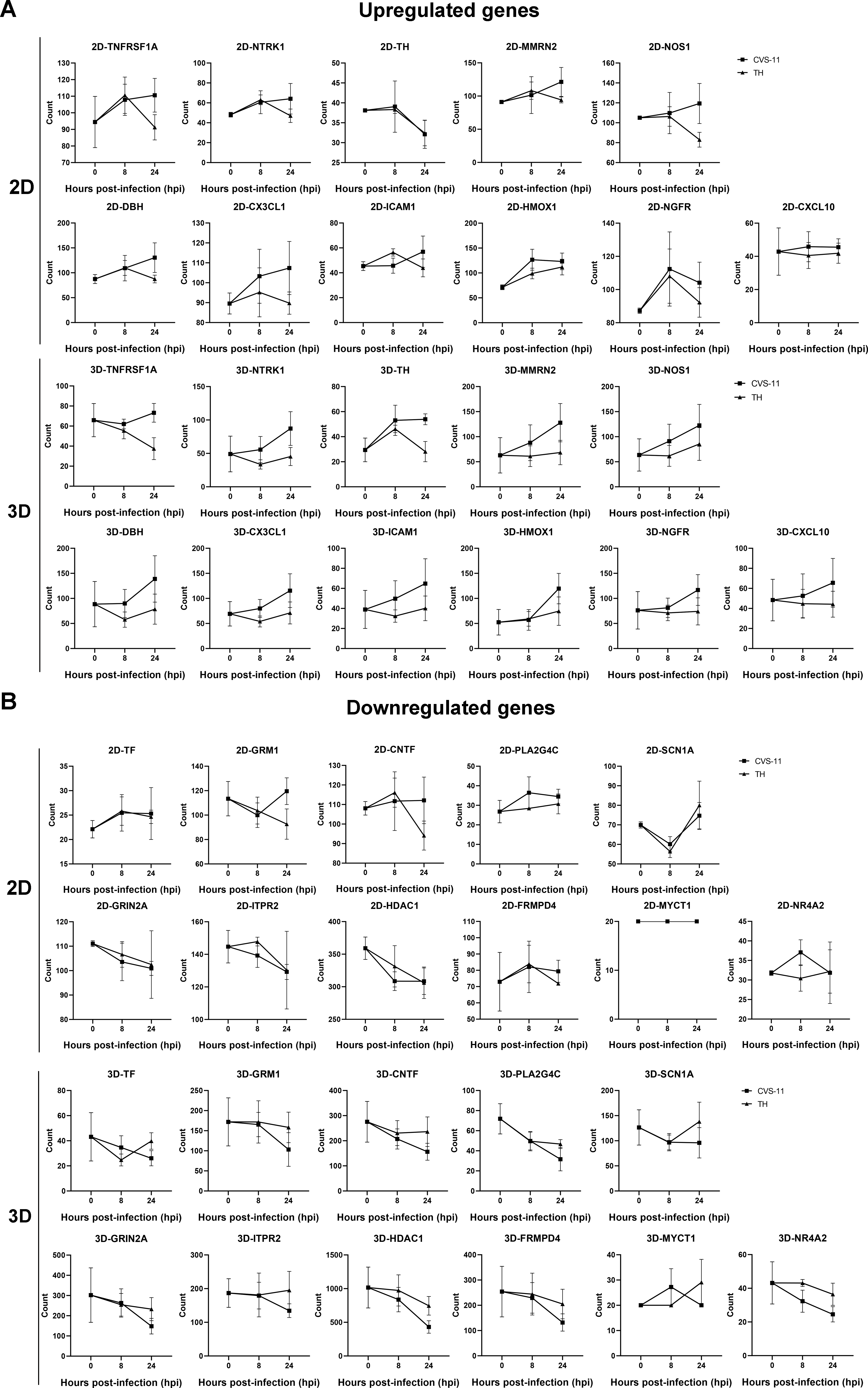

Supplement: Supplementary Figure 9 — NanoString normalized count data of differentially expressed genes. Samples were infected with RABV-TH and RABV-CVS-11 at MOI of 0.5 for 24 h. Total RNA was collected for both 2D and 3D culture samples at 0, 8, and 24 hpi via TRIzol reagent. One hundred nanograms of the purified total RNA were analyzed using the nCounter Analysis System. Normalized count data of selected 22 differentially expressed genes included (A) 11 upregulated genes and (B) 11 downregulated genes. [file Image_9.tif]
